# A natural variation within duplicated *AsWRKY49‐D2* drives the subgenomic functional divergence of homeologs in salt response of allohexaploid oats

**DOI:** 10.1111/jipb.70171

**Published:** 2026-02-09

**Authors:** Cailian Du, Yange Yun, Wenjia Li, Xiaolu Wu, Xingyu Liu, Minghao Li, Yingying Li, Shuhui Wang, Wei Li, Qiang He, Zhizhong Gong, Huilong Du, Qingbin Sun

**Affiliations:** ^1^ College of Life Sciences, Institute of Life Science and Green Development Hebei University Baoding 071000 China; ^2^ Hebei Basic Science Center for Biotic Interaction Hebei University Baoding 071000 China; ^3^ State Key Laboratory of Plant Environmental Resilience, College of Biological Sciences China Agricultural University Beijing 100094 China

**Keywords:** allohexaploid oat, AsWRKY49, natural variation, salt stress, subgenomic functional divergence

## Abstract

Salt stress is a major abiotic constraint limiting global crop production. Oat (*Avena sativa* L.), an allohexaploid cereal renowned for robust stress tolerance, remains poorly understood in terms of the molecular mechanisms underlying its response to salt stress. Here, we perform transcriptome profiling across multiple developmental stages and tissues of oat under salt stress, and construct the co‐expression regulatory network to identify salt tolerance‐associated gene modules. Notably, 10 salt‐responsive transcription factor (SRTF) families with dynamic expression patterns are identified as core regulators, showing extensive subgenomic functional divergence, characterized by subgenome‐dominant expression, as well as subgenome‐specific duplication or loss events. Further integration with a genome‐wide association study (GWAS) of the germination rate under salt stress in 225 oat accessions identified a 3‐bp InDel variation within the duplicated gene *AsWRKY49‐D2*, which specifically modulates its expression by facilitating binding of the TF *AsZAT18*, with *AsWRKY49‐D2* further mediating oat salt tolerance through targeted regulation of *AsSOS2* and *AsSOS3*. Intriguingly, the salt‐tolerant allele of *AsWRKY49* is scarcely distributed in Chinese oat accessions, highlighting its considerable potential for breeding application. These results shed light on the regulatory mechanisms underlying oat salt tolerance, providing valuable information for exploring salt tolerance genes and breeding new salt‐tolerant oat varieties.

## Graphical abstract

Transcriptome analysis of oats under salt stress identified ten important salt‐responsive transcription factor families, which show extensive functional divergence among three oat subgenomes. Further integration with a genome‐wide association study under salt stress identified the *AsWRKY49‐D2* gene, whose elite haplotype holds substantial potential for genetic improvement of salt‐tolerant oats

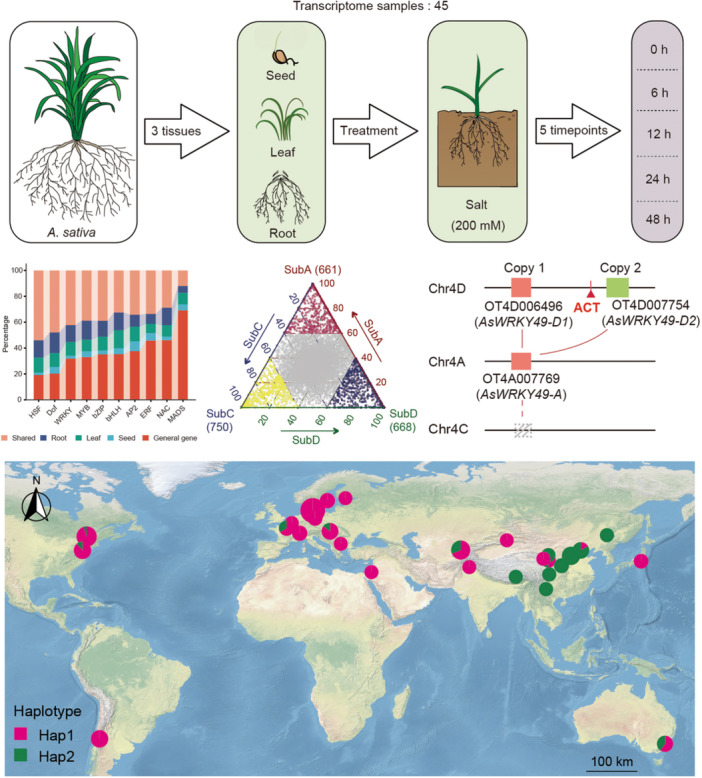

## INTRODUCTION

Salt stress, a major abiotic stress constraining crop productivity on over 20% of global cultivated areas, not only imposes osmotic and ionic stress on plants but also triggers secondary stresses, particularly oxidative stress. Furthermore, this stress has frequently occurred in recent years and is becoming increasingly severe (Zhang et al., 2022a; Liang et al., 2024). Oat (*Avena sativa* L.) serves both as an economically valuable cereal and as a key forage for livestock worldwide. Notably, it shows robust stress tolerance (e.g., drought, cold, and saline–alkali tolerance) and can be cultivated across diverse soil types and marginal regions with adverse growing conditions (Han et al., 2015; Bekele et al., 2018). Therefore, identification of salt‐tolerance genes in oats could facilitate the genetic improvement of salt tolerance in oats as well as major food crops such as wheat and rice, thereby enhancing the utilization of saline–alkali soils. However, to date, the genetic basis underlying oat salt responses remains poorly understood, highlighting an urgent need to elucidate the molecular mechanisms governing salt tolerance and identify key salt‐responsive genes in oats.

Polyploids are arguably the most important force in species diversification, gene function innovation, and important traits' formation (Jackson and Chen, 2010). As allohexaploids, genes from one subgenome in oat are preferentially retained at a higher expression level than those from other subgenomes, namely, “subgenome dominance”, which is also a common phenomenon in other polyploid plants (Li et al., 2014; Wang et al., 2016; Zhang et al., 2025b). For example, of the three *TaSPL6* homeologs on A, B, and D subgenomes in wheat, only *TaSPL6‐D* is dominantly expressed in root stelar cells and negatively regulates the salt tolerance gene *TaHKT1;5‐D* (Wang et al., 2024). Furthermore, gene duplication or loss event may also occur in a specific subgenome (Woodhouse et al., 2010), and duplicated genes tend to have higher expression levels and contribute more to morphological development (Cheng et al., 2012). For instance, in allotetraploid cotton *Gossypium hirsutum*, a duplication of the E3 ubiquitin ligase BB2 on chromosome D05 of the Dt subgenome shows a specific expression pattern during critical cotton fiber development stages (You et al., 2023). Consequently, polyploid plants with various subgenomic functional divergence usually show greater adaptation to extreme environments, allowing them to better survive these disastrous climates (Wang et al., 2022). Thus, in allohexaploid oat, deciphering of subgenomic functional divergence and identification of key differentiated genes could better explain their adaptive mechanisms to specific environmental conditions.

Transcription factors (TFs), by binding to local and distal *cis*‐elements of a given gene under different biological contexts, can precisely modulate gene expression, and as such, they regulate essential aspects of plant function (Inukai et al., 2017). Decades of research have shown that various TFs play an extremely important role in regulating plant responses to abiotic stresses (Liu et al., 2024; Xiong et al., 2025; Yang et al., 2025; Zhang et al., 2025a). Moreover, extensive research has demonstrated that the subgenomic functional divergence of TFs in polyploid plants plays a crucial role in regulating various biological processes. For example, paleopolyploidy‐duplicated salt tolerance‐conferring TFs *AtDDF1* and *AtDDF2* in *Arabidopsis thaliana* show highly asymmetric expression divergence, with *AtDDF2* losing most ancestral stress responses due to promoter disablement (Lehti‐Shiu et al., 2015); in allohexaploid wheat, TFs such as *NAC*, *bZIP*, and *SPL* are highly expressed in the A subgenome of endosperm at 5–9 d after pollination (DAP), whereas those including *ARF* and *MYB* show dominant expression in the B and D subgenomes of grains at 15–20 DAP (Pei et al., 2023). Overall, research on the subgenomic functional divergence of TFs not only provides a core entry point for analyzing the regulatory mechanism of the fate of redundant genes in the genomes of polyploid species but also helps reveal the molecular code that enables polyploid plants to adapt to environmental stresses.

In this study, we performed transcriptome profiling of three oat tissues across different developmental stages under salt stress, followed by construction of a co‐expression network to identify gene modules tightly associated with oat salt tolerance. Notably, we found that 10 TF families play pivotal roles in oat salt stress response, and salt‐responsive TFs show distinct subgenomic functional differentiation, encompassing subgenome‐dominant expression, as well as subgenome‐specific duplication or loss events. Furthermore, integration with a GWAS analysis of germination rates in 225 oat accessions under salt stress identified a 3‐bp variation within *AsWRKY49‐D2*, a duplicated gene located on chromosome 4 of the D subgenome, that specifically modulates its expression by facilitating binding of a *ZAT* TF. This allele has not been exploited in several major oat production areas such as China, highlighting its breeding potential for salt‐tolerant oats. This study contributes toward a comprehensive understanding of the response mechanism of oats to salt stress and provides excellent gene targets for the genetic improvement of salt tolerance in oats.

## RESULTS

### Transcriptome profile under salt stress across developmental stages and tissues

To identify the optimal NaCl concentration for subsequent assays, we first evaluated the relative germination rates of 17 randomly selected oat accessions under salt stress, and the results demonstrated that treatment with 200 mM NaCl elicited the most pronounced phenotypic variation across these accessions (Figure S1A). Consequently, this concentration was designated as the optimal salt stress treatment for all follow‐up experiments. Subsequently, we further assessed the response of oats to salt stress at the seed germination stage and the seedling stage to gain deeper insights into the dynamic responses of oats to salt stress. Under salt stress, the germination rate, the lengths of plumules and radicles, as well as the plant height of seedlings were all significantly reduced. Simultaneously, the leaves of oat seedlings curled and became pale, with an obvious reduction in chlorophyll content (Figure S1B–E).

**Figure 1:**
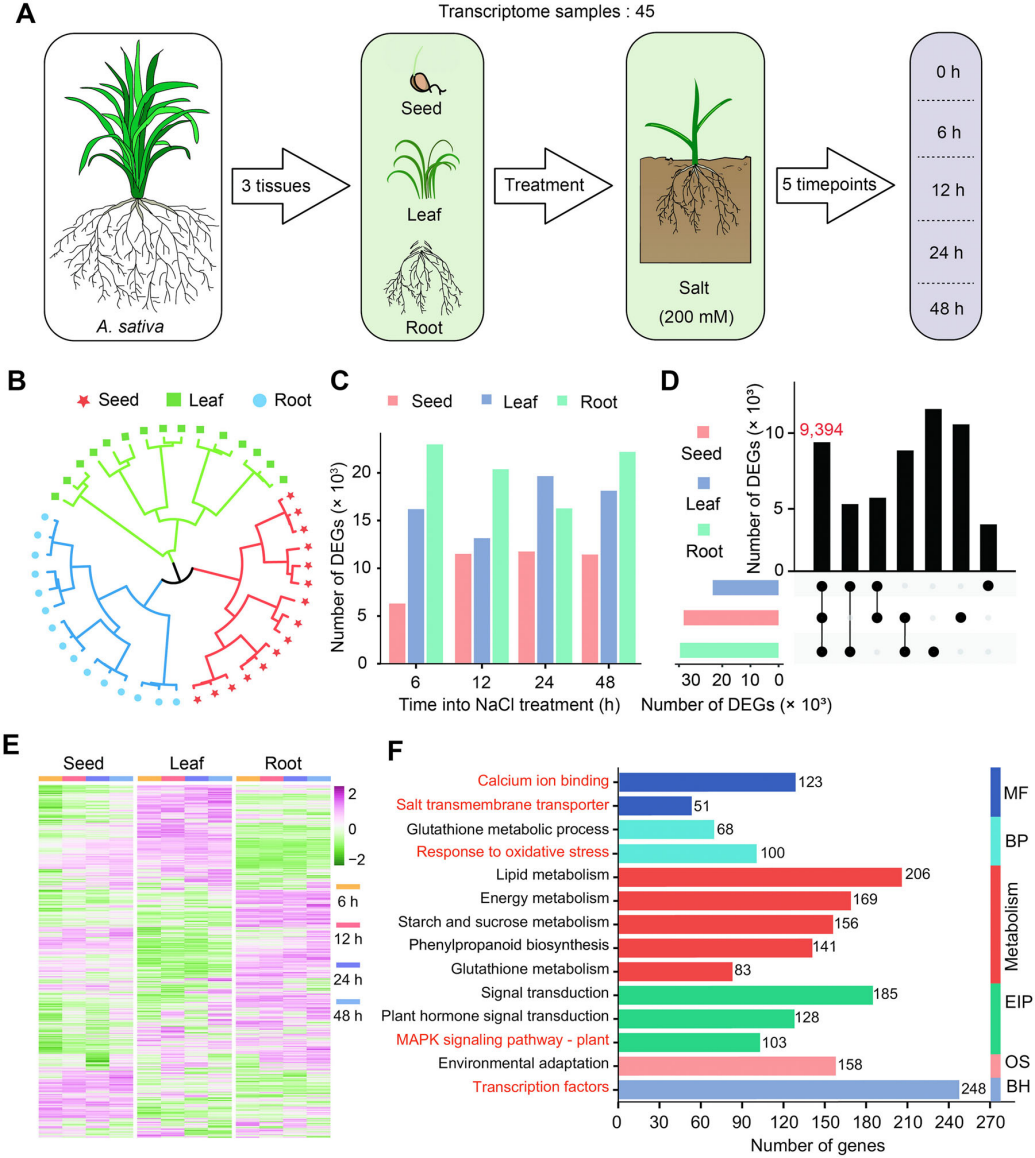
Transcriptome profiling of different tissues from oats under salinity stress **(A)** Diagram representing the workflow of the transcriptome analysis in oats. Seeds were germinated in Hoagland's nutrient solution containing 200 mM NaCl; seedlings (at the two‐leaf and one‐heart stage, grown in Hoagland's nutrient solution for 10 d) were also exposed to 200 mM NaCl and split into roots and leaves. The 0‐h samples were used as controls and came from samples grown in Hoagland's nutrient solution only. **(B)** Hierarchical clustering of all RNA‐seq samples. **(C)** Number of differentially expressed genes (DEGs) identified in each of the oat tissues at each time point relative to the 0‐h time point. **(D)** UpSet plot showing the number of shared and unique salt‐responsive genes in each oat tissue. The red number represents the core DEGs shared among the three tissues analyzed. **(E)** Heatmap representation of expression levels (RNA‐seq) for all 9,394 core DEGs. Heatmap constructed via Z‐score normalization and plotted with the OmicStudio tools (https://www.omicstudio.cn). **(F)** Gene ontology (GO) term enrichment analysis of the core DEGs. The terms in red are closely related to salt stress in plants. BH, brite hierarchies; BP, biological process; EIP, environmental information processing; MF, molecular function; OS, organismal systems.

Then, transcriptome data were obtained from germinating oat seeds (referred to as “Seed”) and the aboveground/underground tissues of oat seedlings (referred to as “Leaf” and “Root”), following salt stress treatment for 0, 6, 12, 24, and 48 h (Figure 1A). A total of 45 transcriptome samples and 306.79 Gb data were generated, with an average mapping rate of 94.68% to the oat reference genome (https://wheat.pw.usda.gov/jb?data=/ggds/oat-ot3098v2-pepsico), indicating the high quality of RNA‐seq libraries (Table S1).

Before proceeding with the identification of differentially expressed genes (DEGs), we performed hierarchical clustering (Figure 1B) and principal component analysis (PCA; Figure S1F) to estimate the similarity between samples. As shown in Figure 1B, biological replicates clustered together globally, with distinct separation among the three tissue‐specific clusters. In addition, the two major principal components in PCA analysis can also well separate these three tissues (Figure S1G), reflecting the good quality and repeatability of our samples.

We then proceeded to identify DEGs by comparing salt‐treated samples with the controls for each tissue at the four time points, yielding a total of 54,390 DEGs (Tables S2, S3). Notably, the number of DEGs (36,995) peaked after 48 h of salt treatment, reflecting a gradual increase in salt‐responsive genes with extended stress duration. Additionally, root tissue contained the highest number of DEGs (35,001), supporting the notion that oat roots are the primary tissue involved in salt stress responses (Figures 1C, S1H). Subsequently, functional characterization of DEGs was carried out through Gene Ontology (GO) and Kyoto Encyclopedia of Genes and Genomes (KEGG) analyses. Common terms shared in three tissues were mainly involved in environmental information processing, the MAPK signaling pathway, ion binding, and kinase activity. Specifically, the unique functional terms in seeds included diterpene biosynthesis, and response to auxin; those unique to leaves were alkaloid biosynthesis, organic nitrogen compound metabolic processes, and regulation of salicylic acid‐mediated signaling pathways; and those specific to roots involved phosphate metabolism and pyruvate metabolism (Figure S1G; Tables S4, S5), indicating that these DEGs may function in a tissue‐specific manner.

We further identified 9,394 shared DEGs among the three tissues, defined as the core salt‐responsive genes (Figure 1D). Clustering heatmap revealed that the expression abundance of these genes varies among different tissues (Figure 1E), demonstrating that oats may respond to salt stress by regulating the tissue‐specific expression of key genes. Functional enrichment analysis of these core salt‐responsive genes showed a strong association with salt response‐related pathways and processes, including ion binding, salt transmembrane transporter activity, response to oxidative stress, the MAPK signaling pathway, and TF activity. Notably, the TF functional category contained the largest number of DEGs (248) (Figure 1F; Tables S4, S5), indicating that TFs may play an extremely critical role in the process of oats responding to salt stress.

### Dynamic expression of transcription factors in oats under salt stress

**Figure 2:**
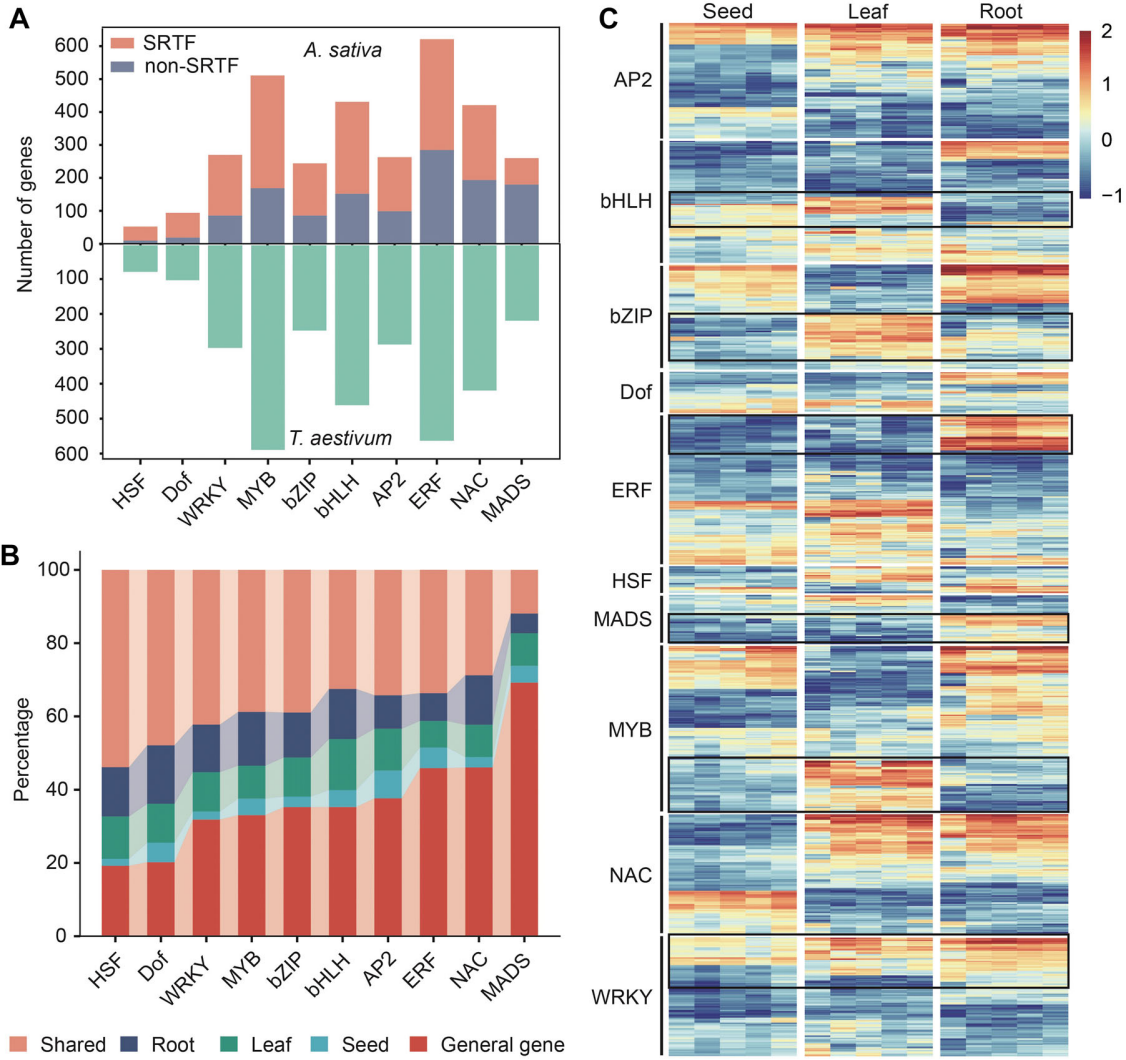
Dynamic expression patterns of salt‐responsive TF genes in oat **(A)** Number of transcription factor (TF) genes belonging to the indicated families in oats and wheat. SRTF, salt‐responsive TF genes; non‐SRTF, not salt‐responsive TF genes. **(B)** Relative proportions of differentially expressed TF genes belonging to the indicated families in three oat tissues. **(C)** Heatmap representation of expression levels (RNA‐seq) for salt‐responsive TF genes in oats. The black rectangles highlight the TF genes that are specifically expressed in single tissue. Heatmap constructed via Z‐score normalization and plotted with the OmicStudio tools (https://www.omicstudio.cn).

Transcription factors (TFs) are master regulators of gene networks and therefore have been targets for genetic improvement in crops (Bhoite et al., 2025). To further investigate the role of TFs in the salt stress response of oats, we systematically identified salt‐responsive TFs (SRTFs) and revealed that *MYB*, *WRKY*, *bHLH*, *AP2*, *ERF*, *NAC*, *MADS*, *Dof*, *bZIP*, and *HSF* represent the major TF families involved in this process. Meanwhile, a similar number of these 10 TF families have also been identified in wheat (Chinese Spring v2.1) (Figure 2A; Table S6), implying that these TF families may show functional conservation and evolutionary relatedness among gramineous crops. Among them, the *MYB* family contained the highest number of SRTFs (342), followed by *ERF* family (336), whereas the *HSF* family harbored the fewest (42) (Figure 2A). We also compared the proportion of SRTFs among the 10 TF families, with values ranging from 30% to 80%. *HSF*, *Dof*, and *WRKY* had the highest proportions (80.77%, 79.79%, and 68.15%, respectively), followed by *MYB* (66.93%), while *MADS* had the lowest (30.77%) (Figure 2B). Overall, 59.33% of oat TFs responded to salt stress, compared with only 41.47% of all genes, further highlighting the key role of TFs in oat salt stress responses (Figure S2A). These results suggest that these TF families may show distinct functional roles in mediating salt responses, and a greater abundance of SRTFs may underpin more elaborate regulatory mechanisms.

Additionally, we compared the number and proportion of SRTFs across the three oat tissues. In general, both the number and the proportion of all SRTFs in leaves and roots were higher than those in seeds (Figure S2B). Most SRTFs were ubiquitously expressed among the three tissues, whereas a substantial number, such as members of the *MADS* (61.25%), and *bHLH* (50.80%) families, showed tissue‐specific expression patterns (Figure 2B). Notably, most SRTFs displayed root‐specific expression, such as those belonging to the *Dof* (6.67% in seed, 13.33% in leaf, and 20.00% in root), *MYB* (6.73% in seed, 13.45% in leaf, and 21.93% in root), *NAC* (5.29% in seed, 16.30% in leaf, and 25.11% in root), and *WRKY* (3.26% in seed, 15.76% in leaf, and 19.02% in root) families, while the *AP2* family (12.20% in seed, 18.29% in leaf, and 14.63% in root) was characterized by more leaf‐specific expression. The expression profiles of some SRTFs showed high consistency between leaves and roots (seedling stage), including members of the *bHLH* (7.17% in seed, 21.51% in leaf, and 21.15% in root), *HSF* (2.38% in seed, 14.29% in leaf, and 16.67% in root), and *bZIP* (4.43% in seed, 16.46% in leaf, and 18.99% in root) families (Figures 2B, S2C; Table S7). Moreover, the heatmap depicting the expression profiles of 10 TF families in oats under salt stress also showed their distinct expression patterns across the three tissues. For instance, certain genes in the *MYB*, *bHLH*, and *bZIP* families show leaf‐specific expression pattern, while *ERF*, *WRKY*, and *MADS* families have higher expression levels in roots (Figure 2C). These results indicate that these 10 TF families play a crucial role in oat salt stress response, and their distribution and expression patterns show high degree of dynamism.

### Construction of a salt‐responsive co‐expression regulation network in oats

**Figure 3:**
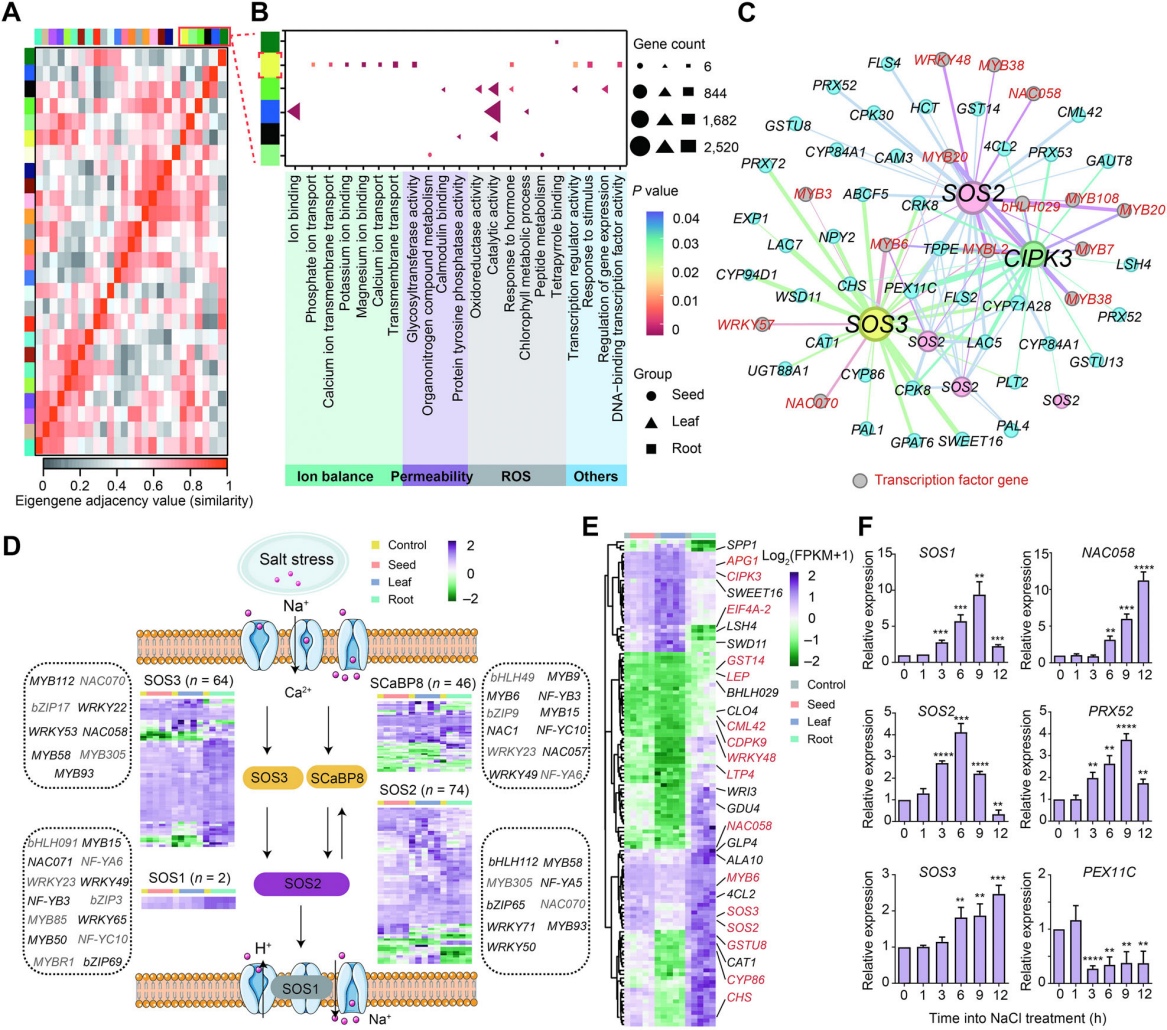
Salt stress‐responsive co‐expression network in oats **(A)** Heatmap showing the extent of correlation among the 26 gene co‐expression modules identified by WGCNA. The red rectangle in the upper right corner highlights modules comprising differentially expressed genes (DEGs) that are dominantly expressed in a specific tissue. The color scale (0–1, unitless) shows the adjacency between module eigengenes, where red (high value) indicates high expression similarity and blue (low value) indicates low similarity. **(B)** GO term and Kyoto Encyclopedia of Genes and Genomes (KEGG) pathway enrichment analysis of the DEGs in the modules highlighted in red in **(A)**. The dashed red box highlights the yellow module, which is mainly related to the function of ion homeostasis under salt stress. **(C)** Co‐expression network of *SOS* genes in the yellow module. Lines between nodes (genes) indicate the weighted Pearson's correlation coefficients for each gene pair. Red and blue nodes denote salt‐responsive TF genes and other protein‐coding genes, respectively. **(D)** List of key salt‐responsive components in the SOS pathway. The heatmaps represent the expression levels of key members in the SOS pathway under salt stress. The genes in the boxes are differentially expressed TF genes that are co‐expressed with SOS signaling pathway genes. Gene names in black are from well‐known salt‐responsive TF genes, while gene names in gray denote those with uncharacterized functions in salt stress. **(E)** Heatmap representation of expression levels for genes that are co‐expressed with components of the SOS pathway. Genes known to be associated with salt tolerance are highlighted in red, while those with uncharacterized roles in salt response are in black. **(F)** RT‐qPCR analysis validating the changes in expression levels of known and uncharacterized salt‐responsive genes in the identified networks and pathways. These experiments were performed using root tissues.

To further investigate the transcriptional changes and gene co‐expression relationships in oats under salt stress, weighted gene co‐expression network analysis (WGCNA) was performed to cluster all DEGs into 26 distinct modules (Figures 3A, S3A, B). Among them, the MEturquoise module contained the largest number of DEGs (10,210), while the MEdarkorange module had the smallest (41) (Table S8; Figure S3C). Further analysis of the tissue‐specific distribution of DEGs across modules revealed that DEGs derived from seeds were predominantly assigned to the MElightgreen module, those from leaves were distributed across the MEblue, MEgreen, and MEblack modules, while DEGs from roots were primarily enriched in the MEyellow and MEdarkgreen modules (Table S8). GO and KEGG enrichment analyses showed that these genes were mainly involved in ion homeostasis, reactive oxygen species (ROS)‐related processes, osmotic regulation, and stimulus response, all of which belong to the classical regulatory pathways of plant response to salt stress (Figure 3B; Tables S9, S10).

Interestingly, genes in the MEyellow module were primarily enriched in ion homeostasis (Figure 3B), which is extremely critical to salt tolerance in plants. Consistent with this functional enrichment, we identified SOS2 and SOS3, key members involved in the Salt Overly Sensitive (SOS) pathway that plays a dominant role in regulating ion homeostasis, within the MEyellow module, further supporting the reliability of our network (Figure 3C; Table S11). Furthermore, we found that a large number of TFs, such as *MYB*, *NAC*, and *WRKY*, were co‐expressed with SOS pathway components in this module (Figure 3C), suggesting potential transcriptional regulation of the SOS‐mediated ion homeostasis pathway by these TFs. In addition, this module also contains several previously unreported genes (e.g., *SWEET16* and *bHLH029*), which are highly likely to affect oat salt tolerance by regulating ion homeostasis, representing a promising direction for future in‐depth research.

We further identified major components of the SOS pathway among the DEGs, including two *AsSOS1*, 74 *AsSOS2*, 64 *AsSOS3*, and 46 *AsScaBP8*, with the majority of these genes showing root tissue‐specific expression patterns (Figure 3D). Meanwhile, we also identified SRTFs co‐expressed with SOS pathway genes. Notably, numerous homologous genes of these SRTFs had been reported in other plant species to be involved in regulating plant salt stress responses, such as *MYB112*, *NAC058*, *WRKY65*, *NF‐YB3*, and *bHLH112* (Liu et al., 2015; Lotkowska et al., 2015; Yan et al., 2019; Min et al., 2020; Zhang et al., 2024), further confirming the accuracy and biological relevance of the network. Overall, as shown in Figure 3E, a large number of genes among the MEyellow module, all *AsSOS* genes, and all SRTFs co‐expressed with *AsSOS* were reported to be associated with plant salt tolerance, and they also showed distinct tissue‐specific expression patterns (Figure 3E). To further validate the reliability of this salt tolerance‐related module, we randomly selected six genes for RT‐qPCR assays, and the results showed that all genes responded to salt treatment (Figure 3F), implying that this module and the TFs within it were likely related to salt tolerance. Collectively, these results indicate that we have identified a co‐expression module associated with ion homeostasis in oats, and TFs may play a crucial regulatory role in this module.

### Subgenomic functional divergence of salt‐responsive TFs in allohexaploid oats

**Figure 4:**
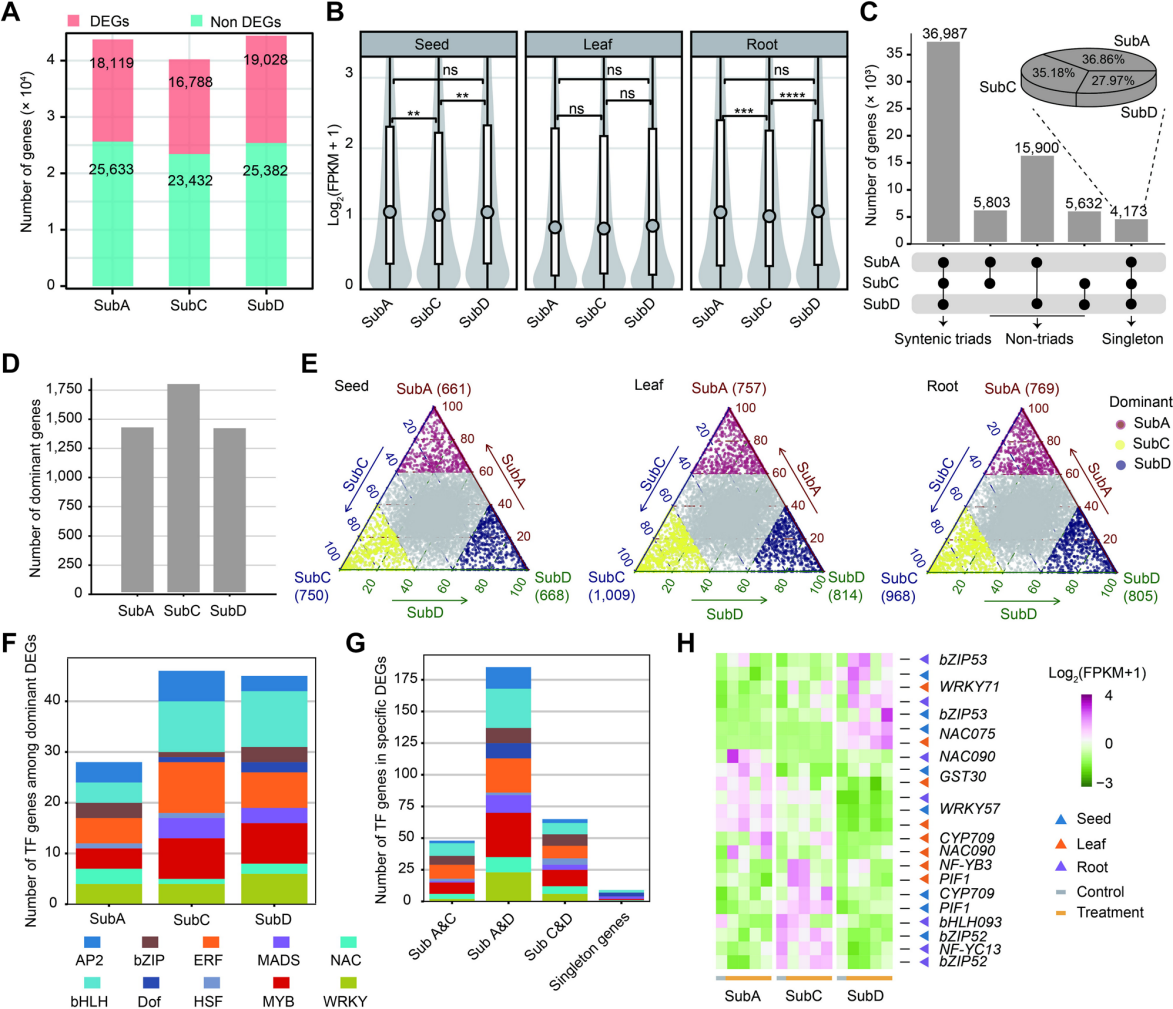
Subgenomic functional divergence of salt‐responsive TF genes in oat **(A)** Number of genes and salt stress‐responsive differentially expressed genes (DEGs) in each subgenome of oats. **(B)** Violin plots showing the expression levels of DEGs in each subgenome of oats across three tissues. Asterisks indicate significant differences, as determined by a Student's *t*‐test (**P < 0.01; ***P < 0.001; ****P < 0.0001; and ns, not significant). **(C)** Number of genes existing as singletons, dyads, or syntenic triads across the three subgenomes of oats. **(D)** Number of dominantly expressed genes in each subgenome of oat across three tissues. **(E)** Ternary plot illustrating the expression bias of homeolog triplets among three subgenomes of oats. Each circle represents a homeologous triad. The Euclidean distance between the triad and its three vertices reflects the relative expression levels of each homeolog under salt stress. Homeologs with higher expression in the A subgenome relative to the other two subgenomes were defined as A‐dominant homeologs; the same definition applies for genes dominantly expressed in one of the other two subgenomes. The gray area in the center of the ternary plot represents homeologs with balanced expression across the three subgenomes. Heatmap was constructed via Z‐score normalization and plotted with the OmicStudio tools (https://www.omicstudio.cn). **(F)** Number of dominantly expressed TF genes within homeolog triads, sorted by TF gene family. **(G)** Number of divergently expressed TF genes not represented by homologous triads (diads and singletons). **(H)** Heatmap representation of expression levels (RNA‐seq) for differentially expressed TF genes across the three subgenomes of oats.

As an allohexaploid plant, oats are considered to have strong environmental adaptability, and subgenomic functional divergence may be an important molecular basis for its broad adaptability (Jia et al., 2025). To explore the role of subgenomic functional divergence in oat salt stress responses, we first analyzed the expression differences of salt‐responsive genes across the A, C, and D subgenomes. These subgenomes harbor 43,752, 40,220, and 44,410 protein‐annotated genes, respectively, with 18,119, 16,788, and 19,028 salt‐responsive DEGs identified therein (Figure 4A). The expression levels of DEGs are the highest in the D subgenome, followed by subgenomes A and C in the seed tissue (Figure 4B), consistent with the overall expression trends of all genes in each subgenome (Figure S4A), suggesting that the salt‐responsive genes in oats show subgenomic expression divergence.

All 131,157 protein‐annotated genes were further categorized into five major homologous groups, including triad genes, subgenome‐specific single‐copy genes, and genes present in any two of the subgenomes (Figure 4C). A total of 12,329 triad gene pairs were identified in oats (Table S12), and the C subgenome harbored more dominantly expressed triad genes than the A and D subgenomes (Figure 4D). Furthermore, the C subgenome also contained the most dominantly expressed triad salt‐responsive genes relative to the other two subgenomes (Figure S4B). Among the three tissues, the number of seed‐dominant genes was lower, while the numbers of leaf‐ and root‐dominant genes were higher (Figure 4E). Similarly, salt‐responsive genes shared the same tissue distribution pattern (Figure S4B). In the other four homologous groups, we found that the “Subgenome A and D” category contained the most salt‐responsive genes, whereas the “subgenome‐specific single‐copy genes” group had the fewest (Figure S4C).

We further identified SRTFs across these five major homologous groups, with distinct distribution patterns observed among subgenomes. In the “triad genes” group, the number of SRTFs in subgenomes C and D was similar, while the subgenome A harbored significantly fewer SRTFs (Figure 4F; Tables S13–S15). In the other four homologous groups, the “Subgenome A and D” category contained the most SRTFs (Figure 4G; Tables S16–S18), consistent with the distribution pattern of salt‐responsive DEGs in this category (Figure S4C). Moreover, the WRKY, MYB, bHLH, and ERF families were the most enriched SRTFs in these five major homologous groups (Figure 4F, G), indicating that these SRTFs play pivotal roles in mediating subgenomic functional divergence during oat salt stress responses. For instance, the expression heatmap revealed that *bZIP53*, *NAC075*, and *WRKY71* were specifically highly expressed in the D subgenome under salt stress; *NF‐YC13*, *bHLH093*, and *bZIP52* were specifically highly expressed in the C subgenome; whereas *WRKY57* and *NAC090* showed specific high expression in the A subgenome (Figure 4H). Overall, these findings demonstrate that salt‐responsive genes in oats, particularly the SRTFs, show extensive subgenomic functional divergence, providing a new perspective for elucidating the mechanisms underlying oat salt tolerance.

### A natural variation near *AsWRKY49‐D2* drives functional divergence among three homeologs and enhances oat salt tolerance

**Figure 5:**
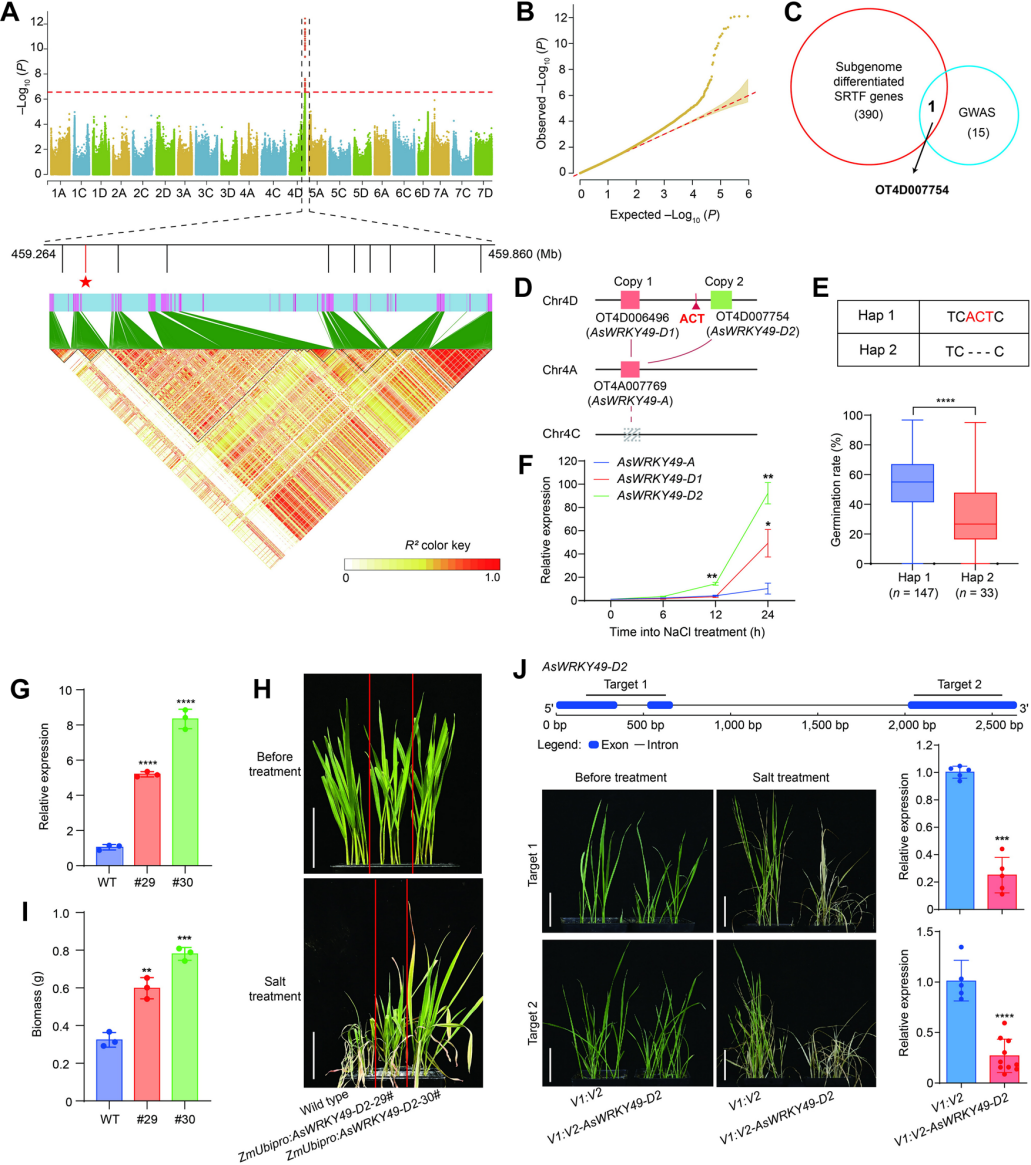
**The differentiated TF gene** ***AsWRKY49‐D2*** **positively regulates salt tolerance in oat** **(A)** Manhattan plot of the genome‐wide association study (GWAS) for salt tolerance in oat. The red dashed line indicates the genome‐wide significance threshold (*P* = 2.76 × 10^−7^). The black dashed box denotes the peak region associated with the germination rate on chromosome 4D. The zoomed‐in panel shows the positions of 10 expressed candidate genes and the linkage disequilibrium (LD) heatmap of single‐nucleotide polymorphisms (SNPs) within this region, with *AsWRKY49‐D2* highlighted in red and with a star. **(B)** Quantile–quantile (QQ) plot for GWAS of the germination rate in oats. **(C)** Venn diagram showing the extent of overlap between salt‐responsive TF (SRTF) genes with subgenomic functional divergence and genes within GWAS peak. **(D)** Distribution of *AsWRKY49‐D2* and its two homeologs in the oat subgenomes. **(E)** Top, nucleotide sequences of Haplotype 1 (Hap1: TCACTC) and Haplotype 2 (Hap2: TC‐‐‐C) of *AsWRKY49‐D2*, classified by the InDel_459300121; bottom, boxplot showing the germination rates in oat accessions harboring Hap1 (*n* = 147) or Hap2 (*n* = 33). Asterisks indicate significant difference, as determined by a Student's *t*‐test (****P < 0.0001). **(F)** Relative transcript levels of *AsWRKY49‐A*, *AsWRKY49‐D1*, and *AsWRKY49‐D2* in oat roots after salt treatment for 0, 6, 12, or 24 h. **(G)** Relative *AsWRKY49‐D2* transcript levels in wild‐type (WT) and *ZmUbipro:AsWRKY49‐D2* transgenic overexpression lines. **(H)** Salt tolerance phenotypes of WT and *AsWRKY49‐D2* overexpression lines. Photographs were taken prior to salt stress treatment and 10 d into salt stress. Scale bar, 5 cm. **(I)** Biomass of WT plants and *AsWRKY49‐D2* overexpression lines. **(J)** Salt tolerance phenotypes of *AsWRKY49‐D2* knockdown lines by virus‐induced gene silencing (VIGS) using the TRV system. Top, diagram of the *AsWRKY49‐D2* locus showing the two gene fragments (target) used for VIGS; bottom left, representative photographs of *AsWRKY49‐D2* knockdown lines and control plants before and after salt treatment for 10 d; and bottom right, relative *AsWRKY49‐D2* transcript levels in the plants shown in this panel. Scale bar = 5 cm. Values in **(G**, **I**, and **J)** are means ± *SD* from at least three independent experiments; asterisks indicate significance, as determined by a Student's *t*‐test (**P < 0.01; ***P < 0.001; ****P < 0.0001).

To further investigate the genetic basis of oat salt stress response, we also quantified the germination rates of 225 oat accessions under salt stress (Figure S5A). Using a mixed linear model that corrected for population structure and kinship (*P* = 2.76 × 10^−7^), a GWAS was performed using filtered SNPs from resequencing data (Li et al., 2025), and a linkage‐disequilibrium (LD) block was identified on chromosome 4D (Figure 5A, B). This locus harbored 15 high‐confidence candidate genes, and transcriptome analysis revealed that only 10 of these genes were expressed in any of the tissues (Figure 5A; Table S19). Surprisingly, the OT4D007754 gene was also involved in the subgenome‐differentiated SRTFs (Figure 5C), and belonged to the “Subgenome A and D” group, with no homologous counterpart detected in the C subgenome (Figure 5D), suggesting that it shows subgenomic functional divergence and may play a role in regulating oat responses to salt stress. It encoded a WRKY TF and was referred to as *AsWRKY49‐D2* according to its chromosome location and the evolutionary relationship with its ortholog in *Arabidopsis thaliana* (Figure S5B).

**Figure 6:**
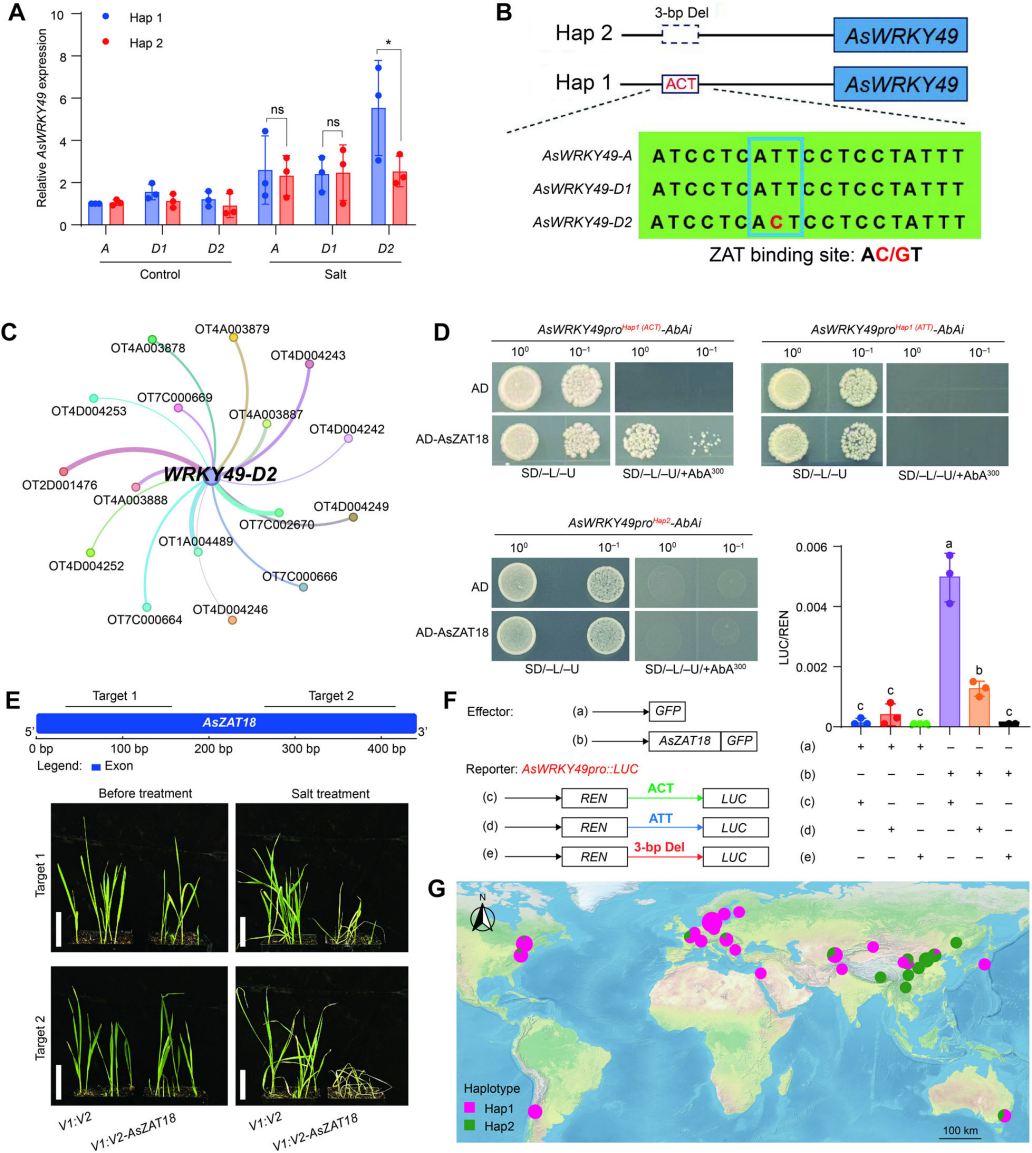
**AsZAT18 specifically regulates the expression of differentiated** ***AsWRKY49‐D2*** **(A)** RT‐qPCR analysis validating the expression levels of *AsWRKY49‐A*, *AsWRKY49‐D1*, and *AsWRKY49‐D2* in randomly selected oat accessions carrying Hap1 or Hap2 and grown under normal or salt stress conditions. Values are means ± *SD* from at least three independent experiments. Asterisks indicate a significance difference, as determined by a Student's *t*‐test (*P < 0.05; ns, not significant) **(B)** Diagram of *AsWRKY49* showing the 3‐bp InDel present in its promoter and the sequence variation in the 3‐bp insertion. “AC/GT” represents the binding site of ZAT transcription factors. **(C)** Identification of ZAT transcription factor genes that are co‐expressed with *AsWRKY49‐D2* in the MEyellow module. Lines indicate the weighted Pearson's correlation coefficients for each gene pair. **(D)** Yeast one‐hybrid (Y1H) assay showing that AsZAT18 specifically binds to the *AsWRKY49* ^*Hap1(ACT)*^, but not to the *AsWRKY49* ^*Hap1(ATT)*^ promoter or to the *AsWRKY49* ^*Hap2*^ promoter. **(E)** Representative photographs showing the salt tolerance phenotypes of WT and *AsZAT18* knockdown lines by VIGS. Top, diagram of the *AsZAT18* locus showing the two gene fragments used for VIGS; bottom, photographs of WT and *AsZAT18* knockdown lines before and after salt treatment for 10 d. Scale bar = 5 cm. **(F)** Diagrams of the constructs used for a dual‐luciferase reporter assays of promoter fragments from different *AsWRKY49* genotypes, with the *AsZAT18‐GFP* vector or empty vector as the effector. *Renilla* luciferase (REN) was included as an internal control. Data represent means ± *SD* with *n* = 3, and different letters indicate the significant differences at the 0.05 level determined by a one‐way ANOVA test. **(G)** Geographic distribution of two *AsWRKY49* haplotypes in each ecological zones of the world. The size of the pie chart reflects the number of oat accessions, with the two haplotypes shown in different colors.

Moreover, using the resequencing data, we identified a 3‐bp insertion/deletion (InDel) with a minor allele frequency (MAF) > 5% at position −1,419 bp in the promoter of *AsWRKY49‐D2* and it showed the strongest association with the germination rate under salt stress (Figure S5C). On the basis of this InDel, these oat accessions were categorized into two haplotype groups: Hap1 and Hap2. Under salt stress, accessions with Hap1 had a higher germination rate than those with Hap2 (Figure 5E). We also randomly selected three accessions in each haplotype group for phenotypic analysis, revealing that Hap1 showed significantly higher seed germination rate, seedling fresh weight, and plant height than Hap2 under salt stress, suggesting that the 3‐bp InDel variation within *AsWRKY49‐D2* may play a key role in regulating oat salt tolerance (Figure S5D, E). *AsWRKY49‐D2* was predominantly expressed in root tissue (Figure S6A). Together with the other two homeologs, their expressions were all upregulated by salt treatment, while *AsWRKY49‐D2* showed the highest expression level (Figure 5F). Additionally, the fusion protein GBD‐AsWRKY49‐D2 showed strong autoactivation activity in yeast, indicating that AsWRKY49‐D2 possesses transcriptional activation activity, and it was localized in the nucleus, both of which are consistent with its property as a TF (Figure 6B, C).

To test whether *AsWRKY49‐D2* is associated with salt tolerance in oat, we generated two independent transgenic oat lines overexpressing *AsWRKY49‐D2*, driven by the maize *Ubiquitin* (*ZmUbi*) promoter (Figures 5G, S6D). While no visible differences were found between wild‐type and *AsWRKY49‐D2* overexpression plants before salt treatment, the *AsWRKY49‐D2* overexpression plants were more tolerant than the wild‐type controls after salt treatment (Figures 5H, I, S6E). In addition, we also generated gene knockdown lines targeting two distinct sites within *AsWRKY49‐D2* using the tobacco rattle virus‐mediated virus‐induced gene silencing (VIGS) system (Figure 5J), and prior to this, we verified the reliability of the VIGS system in oats with the *AsPDS* marker gene (Figure S6F). Compared with the control plants (*V1:V2*), the gene knockdown plants (*V1:V2‐AsWRKY49‐D2*) showed increased sensitivity to salt stress (Figure 5J). Collectively, these results suggest that *AsWRKY49‐D2* shows subgenome functional divergence and positively regulates salt tolerance in oats.

### The natural variation modulates oat salt tolerance by specifically affecting the binding of *AsZAT18*

To elucidate in depth the molecular mechanisms by which *AsWRKY49‐D2* regulates oat salt tolerance through subgenomic functional divergence, we first detected the expression of three homeologs in oat accessions randomly selected from the Hap1 and Hap2 groups. The results showed that while the three homeologs were all upregulated under salt stress, *AsWRKY49‐D2* ^*Hap1*^ showed significantly greater upregulation than *AsWRKY49‐D1* ^*Hap1*^ and *AsWRKY49‐A* ^*Hap1*^ (Figure 6A), suggesting potential key variations in *AsWRKY49‐D2* ^*Hap1*^ underlying its dominant expression. Therefore, detailed sequence analysis of three homeologs revealed a key SNP variation within the 3‐bp InDel of *AsWRKY49‐D2* (Figure 6B), which constitutes a known *cis* element recognized by ZAT TFs (C2H2‐type zinc finger TF) (He et al., 2025). We hypothesize that a *ZAT* TF can specifically bind to the “ACT” motif in *AsWRKY49‐D2* and regulate its expression.

Based on the co‐expression network, we identified all *ZAT* TFs co‐expressed with *AsWRKY49‐D2* (Figure 6C) and performed yeast one‐hybrid (Y1H) assays to screen for *ZAT* TFs capable of binding to the “ACT” motif within *AsWRKY49‐D2*. We identified that OT7C000666 (referred to as *AsZAT18*) could specifically bind to the “ACT” motif in *AsWRKY49‐D2*, but failed to bind to the “ATT” motif or the corresponding sequence in Hap2 (Figures 6D, S7A). Furthermore, this was further validated by an EMSA assay, which demonstrated that the recombinant *AsZAT18* protein bound specifically to the Hap1^ACT^ probe, but showed no binding activity toward the Hap1^ATT^ and Hap2 probes (Figure S7B). *AsZAT18* was also mainly expressed in root tissue, and its expression level increased significantly under salt stress. Additionally, *AsZAT18* was also localized in the nucleus, all of which was consistent with the expression pattern of the *AsWRKY49‐D2* (Figure S7C–E).

To further explore the roles of *AsZAT18* in salt tolerance, we constructed gene knockdown lines targeting two sites within *AsZAT18* via the VIGS system (Figure 6E). Both targets decreased *AsZAT18* expression (Figure S7F), and compared with the control plants (*V1:V2*), *V1:V2‐AsZAT18* plants showed increased sensitivity to salt stress (Figure 6E), suggesting that *AsZAT18* also positively regulates oat salt tolerance. We also examined the effect of *AsZAT18* on *AsWRKY49‐D2* expression and found that the expression of *AsWRKY49‐D2* was downregulated in *V1:V2‐AsZAT18* plants (Figure S7G). Moreover, we conducted a dual‐luciferase (LUC) assay in tobacco leaves, and the results showed that the *AsZAT18‐GFP* effector significantly enhanced the relative LUC activity driven by the *AsWRKY49* ^*Hap1(ACT)*^ promoter (Figure 6F). Additionally, it should be noted that the *AsZAT18*‐GFP effector can also slightly enhance the LUC activity driven by the *AsWRKY49* ^*Hap1(ATT)*^ promoter, which may be attributed to the presence of other TFs capable of recognizing and binding to this type of promoter.

### *AsWRKY49‐D2* mediates salt tolerance in oats through targeted regulation of key members in the SOS pathway

Co‐expression network analysis revealed that *AsWRKY49‐D2* was also assigned to the MEyellow module, previously shown to harbor the key SOS pathway components *SOS2* and *SOS3* (Figure 3C). Thus, we hypothesized that the *AsWRKY49‐D2* may influence oat salt tolerance by regulating *SOS* expression, and performed the Y1H assay to verify the binding of *AsWRKY49‐D2* to the promoters of *AsSOS* genes. The results showed that *AsWRKY49‐D2* can bind to the promoters of both *AsSOS2* (the protein encoded by gene ID OT4A003760) and *AsSOS3* (the protein encoded by gene ID OT1A006486), and sequence analysis revealed that the promoters of these two genes indeed contain multiple conserved W‐box motifs (TTGACC/T) (Figure S8A, B). We further detected the expression of *AsSOS2* and *AsSOS3* in the *AsWRKY49‐D2* overexpression plants and *V1:V2‐AsWRKY49‐D2* gene knockdown plants. The results showed that overexpression of *AsWRKY49‐D2* significantly upregulated the expression levels of *AsSOS2* and *AsSOS3* (Figure S8C), whereas knockdown of *AsWRKY49‐D2* in *V1:V2‐AsWRKY49‐D2* plants suppressed their expression (Figure S8D, E). Furthermore, we analyzed the Na^+^ and K^+^ contents in the shoot tissues of wild‐type and *AsWRKY49‐D2* overexpression plants following treatment with 200 mM NaCl. Under the salt stress condition, we observed that the overexpression plants accumulated significantly lower levels of Na^+^ and higher levels of K^+^ in shoots, resulting in a lower shoot Na^+^/K^+^ ratio compared with the wild type (Figure S8F–H). This finding suggests that *AsWRKY49‐D2* may contribute to the maintenance of ion homeostasis in oats by regulating the expression of *AsSOS2* and *AsSOS3*, thereby modulating the salt tolerance in oats.

### The elite genotype of *AsWRKY49‐D2* holds significant promise for salt‐tolerant oat breeding in China

To optimize the utilization of elite haplotype genes, we analyzed the global distribution of the two haplotypes of *AsWRKY49*. Our results revealed that over 80% of the Hap1 group is distributed across Europe, the Americas, Oceania, and Central and Western Asia. In contrast, more than 85% of oat accessions in China belong to the Hap2 group, with the Hap1 group concentrated in Xinjiang and Qinghai provinces (Figure 6G; Table S20). This distribution pattern is likely associated with the extensive saline–alkali lands in these two regions and the artificial selection exerted during the long‐term cultivation of oats. Therefore, this finding indicates that the Hap1 genotype of *AsWRKY49* holds substantial application potential for the genetic improvement of salt tolerance in oats, as well as in major food crops such as wheat and rice in China.

## DISCUSSION

Oats, renowned for their rich nutritional value, high yield potential, and strong stress tolerance, have emerged as a widely cultivated, versatile dual‐purpose crop globally, and they thrive on diverse soil types even in harsh environments (Yang et al., 2023). However, few studies have reported changes in the physiological phenotypes, transcriptomes, and metabolomes of several oat cultivars under saline–alkali stress (Wu et al., 2018; Xu et al., 2021; Zhang et al., 2022b), and the molecular mechanisms underlying oat's response to salt stress remain largely elusive. In this study, we performed transcriptome analysis of three oat tissues under salt stress (Figure 1), with a focus on dynamic changes in diverse SRTF families (Figure 2), and constructed a co‐expression network (Figure 3). Further analysis found the SRTFs show extensive subgenomic functional divergence (Figure 4). Combining GWAS, a key SNP variation within the duplicated gene *AsWRKY49‐D2* was identified, specifically modulating its expression by facilitating binding of *AsZAT18* (Figures 5, 6). Our study is the first to explore the molecular mechanisms underlying oat salt stress response from the perspective of subgenomic functional divergence and identified a divergent gene *AsWRKY49‐D2*, partially filling the gap in molecular mechanism research of oat salt tolerance.

Subgenomic functional divergence is a widely observed phenomenon in polyploid plant genomes, such as in monkeyflower (Edger et al., 2017), cotton (Yoo et al., 2013), and switchgrass (Lovell et al., 2021). Oats are allohexaploid composed of A, C, and D subgenomes, and a previous study revealed a degree of subgenomic functional differentiation in hexaploid oats and within the *Avena* genus (Zhang et al., 2025b). In this study, we also found that the salt‐responsive genes in oats also show extensive subgenomic functional divergence (Figure 4). The dominantly expressed salt‐responsive genes were most abundant in the C subgenome; however, the expression level of the C subgenome is the lowest (Figures 4B, F, S4B). We believe there may be several reasons for this. When there is redundancy in salt‐responsive genes in a specific subgenome, the expression of these genes will be downregulated to maintain the overall gene dosage balance, avoiding the burden on cellular metabolism. The salt‐responsive genes or TFs in this subgenome may show higher levels of DNA methylation or histone repressive modifications, directly reducing the expression level. In fact, similar phenomena have also been observed in other polyploid species. For instance, salt stress enhances asymmetric expression of the homeologs in allohexaploid wheat roots, and A‐ and D‐dominant homeologs are more related to salt response (Du et al., 2025); in allotetraploid *Nicotiana tabacum*, DNA methylation governs the coordination and differentiation of gene expression between subgenomes (Zan et al., 2025).

The identified *AsWRKY49* gene has been lost from the C subgenome, yet duplicated within the D subgenome, a pattern that may reflect adaptive evolution during oat polyploidization. This is consistent with the well‐documented role of the D subgenome in mediating stress tolerance across *Avena* species (Zhang et al., 2025b). The key locus *qGST4D*, which regulates herbicide tolerance in wild oats, is also located on chromosome 4D, further supplementing the evidence that the D subgenome enhances stress tolerance in oats (Liu et al., 2025a). Furthermore, the analysis of the global distribution of the two haplotypes of *AsWRKY49* showed that the superior salt‐tolerant *AsWRKY49* ^*Hap1*^ allele is only concentrated in Xinjiang and Qinghai provinces in China (Figure 6G), indicating great application potential for improving oat salt tolerance. Targeted improvement of existing main cultivars using excellent genotypes can significantly enhance their core traits such as resistance and yield. For example, introgressing the drought‐tolerant allele *TaNAC071‐A* ^*In‐693*^ into two wheat cultivar (Wanmai33 and Yangmai13) can obviously improve their drought tolerance (Mao et al., 2022); genetic modification of the Gγ subunit AT1 can enhance salt–alkali tolerance in major graminaceous crops, such as rice, maize, wheat, sorghum, and millet (Sun et al., 2023; Zhang et al., 2023); and transferring the *SBRR1‐R* allele into two widely planted *japonica* cultivars, Taigeng 394 (TG394) and Xudao 3 (XD3), can significantly improve the resistance to sheath blight (Feng et al., 2025). Specifically, the saline–alkali lands in China's Bohai Rim region are predominantly characterized by neutral salt accumulation, with NaCl as the major component and a generally stable pH value of around 7 (Shang et al., 2025). Therefore, new salt‐tolerant oat varieties can be developed by incorporating this excellent salt‐tolerant *AsWRKY49* ^*Hap1*^ allele, with promising prospects for cultivation in these severe saline–alkali regions.

## MATERIALS AND METHODS

### Plant materials and growth conditions

The oat cultivar “Baiyan 2” (from Dingxi Academy of Agricultural Sciences) was used in this study. For the phenotypic analysis under slat stress, oat seeds were germinated and cultured in an incubator at 25°C/18°C (day/night), with a 16 h/8 h light/dark photoperiod and 50% relative humidity. For association analysis, the germination rates of 225 oat accessions were obtained in saline–alkali soil (National Technology Innovation Center for Comprehensive Utilization of Saline–Alkali Land, Dongying, Shandong province, China), calculated as the ratio of emerged seedlings to the total number of sown seeds. For the analysis of the global geographic distribution of two haplotypes, the 159 landrace DNA samples sequenced by our lab were used. For the phenotypic analysis of overexpression and VIGS plants, they were grown in a greenhouse at 25°C/18°C (day/night) with a 16 h/8 h light/dark photoperiod. *N. benthamiana* plants were cultured at 22°C with a 12 h /12 h light/dark photoperiod. All phenotypic analyses were treated with a 200 mM salt solution (molar ratio of NaCl:Na_2_SO_4_ = 5:1).

### Transcriptomic sequence and analysis

Transcriptomic sequencing was performed on germinating oat seeds (including radicles and cotyledons) and the aboveground/underground tissues of oat seedlings with 200 mM salt treatment for 0, 6, 12, 24, and 48 h. Three independent replicates were performed and all samples were immediately frozen in liquid nitrogen and stored at −80°C. RNA‐seq libraries were constructed and sequenced by OE Biotech (Shanghai, China) on the Illumina NovaSeq platform (San Diego, CA, USA), and the raw RNA‐seq data have been deposited in the Genome Sequence Archive (BioProject: PRJCA055323). Sequence reads were trimmed using Fastp (v0.20.1). Clean RNA‐seq reads from 45 samples were mapped to the “Oat_OT3098_v2” reference genome using HISAT2 (v2.2.1). Gene expression levels were quantified with StringTie (v2.1.6) based on fragments per kilobase of exon per million mapped fragments (FPKM) values, using the parameters “–G –e –A”. Differentially expressed genes were identified using the DESeq. 2R package (v1.38.3) with default parameters, and then further filtered with a minimum twofold change (|Log_2_FoldChange| ≥ 1) and FPKM > 0.5.

### GO and KEGG enrichment analyses

Genes annotated with Gene Ontology (GO) terms in the “Oat_OT3098_v2” reference genome were used as background genes, totaling 67,072. GO terms and Kyoto Encyclopedia of Genes and Genomes (KEGG) pathways for genes in the “Oat_OT3098_v2” genome were retrieved from corresponding InterPro entries. GO and KEGG enrichment analyses were conducted using TBtools software, with terms and pathways considered significantly enriched at a *P*‐value < 0.05.

### Identification of transcription factors

Protein sequences of the *HSF*, *Dof*, *WRKY*, *MYB*, *bZIP*, *bHLH*, *AP2*, *ERF*, *NAC*, and *MADS* TF families were downloaded from the Arabidopsis Information Resource (TAIR, https://www.arabidopsis.org/) database. These sequences were aligned to the “Oat_OT3098_v2” reference genome using BLASTp with default parameters, and genes with an e‐value < 1e‐5 and similarity > 30% were extracted. Subsequently, hmmsearch (v3.4) was used for further screening, with candidates meeting an e‐value < 1e‐5 retained. Finally, the union of gene sets identified by BLASTp and hmmsearch was taken as the final set of TFs in oats.

### Construction of a salt stress‐responsive gene co‐expression network

To comprehensively evaluate the co‐expression relationships among salt stress‐responsive genes in cultivated oats, gene expression levels were first quantified based on 45 sets of transcriptome data related to oat salt stress tolerance. StringTie (v2.1.6) was used with parameters –G –e –A to calculate FPKM. The R package DESeq. 2 (v1.38.3) was then used to identify DEGs with a minimum twofold expression change (FPKM > 0.2; |Log_2_FoldChange| ≥ 1). Subsequently, the WGCNA package in R was used for data analysis, with 54,390 salt stress‐responsive DEGs as input to construct a scale‐free co‐expression network. In the WGCNA pipeline, we first optimized the adjacency matrix by systematically evaluating the scale‐free topology fit index across a range of β values (1–30). This analysis allowed us to select the optimal soft threshold power of 10 (*R* ^*2*^ = 8.5), which best satisfied the scale‐free network distribution assumption. Co‐expression modules were then constructed using the blockwiseModules function with tailored parameters: Signed topological overlap matrix (TOM) calculation, a module merging cut height of 0.25 to merge modules with high similarity, and a minimum module size of 30 to ensure robust and biologically meaningful module identification. All other parameters were maintained at their default settings. Cytoscape (v3.10.1) and Gephi (v0.10) were used to visualize the network and extract network subsets.

### Identification and classification of homologous genes in the oat subgenome

The Oat_OT3098_v2 gene set was classified according to the origin of its subgenomes (A, C, and D). OrthoFinder (v2.0) with default parameters was used to infer gene families and homologous relationships. In OrthoFinder, homologous genes among subgenomes were treated as distinct classification units. Homologous genes located in conserved collinear regions between subgenomes were defined as syntenic homologous genes. A total of 131,157 genes were annotated in the genome, of which 36,987 are homologous triads, with one copy existing in each of the three subgenomes (A:C:D = 1:1:1). Additionally, 4,173 genes were single copy, with no homologous genes in the other subgenomes (A:C:D = 1:0:0, 0:1:0, or 0:0:1). The remaining homologous genes were defined as non‐triad genes, where one of the subgenomes lacks a homologous gene (A:C:D = *n*:*n*:0, *n*:0:*n*, or 0:*n*:*n*; *n* ≥ 1).

### Genome‐wide association study (GWAS)

For the GWAS on the germination rate of 225 oat accessions under salt stress, high‐quality SNPs (minor allele frequency (MAF) ≥ 0.05, missing rate ≤ 0.1) from 225 oat germplasms were used in an association mapping panel consisting of these oat accessions. The marker–trait associations were analyzed using a mixed linear model in GEMMA software (version 0.98.5), with the results of PCA as covariates. The significance threshold was set at 1 × 10^−4^. Results were summarized and visualized using the CMplot package (version 4.5.1). Linkage disequilibrium analysis of SNPs, structural variations (SVs), and insertions/deletions (Indels) within candidate gene regions was conducted with LDBlockShow software (version 1.40).

### Reverse transcription quantitative PCR (RT‐qPCR) analysis

Total RNA was extracted from plant tissues using Trizol reagent (CWBIO) following the manufacturer's instructions strictly. First‐strand cDNA synthesis was performed with a reverse transcription kit (TIANGEN Biotech, Beijing, China) using the extracted total RNA as the template. Quantitative real‐time PCR (qPCR) was conducted on a LightCycler (Roche, IN, USA) using SYBR Green I dye‐based qPCR enzyme (TIANGEN Biotech, Beijing, China). Primers for target genes and reference genes were designed and synthesized by Sangon Biotech (Shanghai, China). All primers used in qPCR are listed in Table S20. The relative expression levels of target genes were calculated using the 2^−ΔΔCt^ method.

### Subcellular localization

To determine the subcellular localization of *AsWRKY49‐D2* and *AsZAT18* proteins, their coding sequences (CDs) were amplified and ligated into the linearized *pCAMBIA3300‐Ubi–GFP* vector (*EcoR*I), and *ZmUbipro:AsWRKY49‐D2–GFP*, *ZmUbipro:AsZAT18–GFP*, and *ZmUbipro:GFP* vectors were transformed into tobacco leaves. The GFP and fluorescence were visualized using confocal microscopy (LSM900; Carl Zeiss) with excitation wavelengths of 488 and 561 nm, respectively, after incubation at 22°C for 48–82 h. All primers are listed in Table S20.

### Generation of transgenic oat plants overexpressing *AsWRKY49‐D2*

The CDs of *AsWRKY49‐D2* was amplified and ligated into the linearized *pWMB110* vector (*Sac*I), which was then transformed into *Agrobacterium tumefaciens* strain GV3101. Generation of transgenic oat plants overexpressing *AsWRKY49‐D2* was performed as described previously (Shi et al., 2025). The positive seedlings were screened by PCR and RT‐qPCR. All primers are listed in Table S20.

### Yeast one‐hybrid (Y1H) assay

In the *pGADT7*–*pAbAi* system, three tandem copies of all three types of *AsWRKY49* promoters (Hap1(ACT), Hap1 (ATT), and Hap2) were individually synthesized as oligonucleotides and ligated into the *pAbAi* vector (Clontech, Mountain View, CA, USA). Each *pAbAi* vector harboring the constructs was integrated into the genome of the Y1HGold yeast strain (Weidi Biotechnology, Shanghai, China). The coding sequences of *AsZAT18* were PCR‐amplified and cloned into the *pGADT7* vector (Clontech, Mountain View, CA, USA), and the empty *pGADT7* vector was used as a negative control. Transformants were selected on minimal synthetic dropout (SD) medium lacking Leu and Ura. Yeast cells grown in SD/−Leu/−Ura broth were diluted to OD_600_ = 0.5 and plated on an SD/−Leu/−Ura plate with or without AbA as described by the Matchmaker Gold Yeast One‐Hybrid Library Screening System Protocol (Clontech, Mountain View, CA, USA). All plates were incubated at 30°C for 3 d, and colony growth was observed to evaluate the interaction.

In the *pB42AD*–*pLacZi‐2μ* system, the promoter sequences (1,500 bp upstream of the translation start site) of *AsSOS2* and *AsSOS3* were PCR‐amplified and ligated into the linearized *pLacZi‐2μ* vector (*Sal*I). The CDs of *AsWRKY49‐D2* were ligated into the linearized *pB42AD* vector (*EcoR*I), and then different combinations of vectors were co‐transformed into the yeast strain EGY48. Transformants were selected on SD medium laced with Trp and Ura, and then the positive yeast cells were streaked on an SD/−Trp/−Ura plate with X‐Gal as described previously (Sun et al., 2019). All plates were incubated at 30°C for 3–5 d, and colony bluing was used as the criterion to assess whether the interaction occurred. All primers are listed in Table S20.

### Gel mobility shift assay

The CDS of *AsZAT18* was cloned into the *pGEX4T‐1* vector, which harbors a glutathione S‐transferase (GST) tag. The GST‐*AsZAT18* recombinant protein were expressed in the *E. coli* BL21 (DE3) strain and purified using a nickel‐affinity chromatography column. Then, the recombinant protein was incubated with biotin‐labeled and unlabeled Hap1^ACT^ probes, the Hap1^ATT^ probe, and the Hap2‐type probe in binding buffer (Thermo Scientific, Rockford, IL, USA) at room temperature for 25 min. The binding reaction mixtures were separated by 5% native PAGE at 4°C. The interaction between the protein and DNA probes was analyzed by the electrophoretic mobility shift assay (EMSA) using the LightShift Chemiluminescent EMSA Kit (Thermo Scientific, Rockford, IL, USA) following the manufacturer's protocol.

### Virus‐induced gene silencing (VIGS) assay

The VIGS assays were performed as described previously (Zhang et al., 2017; Liu et al., 2025b). In brief, two fragments of different lengths within the ORFs of target genes (*WRKY49‐D2‐1*, *WRKY49‐D2‐2*, *AsZAT18‐1*, and *AsZAT18‐2*) were PCR‐amplified and subcloned into the *TRV2* vector via NC clone (NC Biotech). For infiltration, *A. tumefaciens* carrying *pTRV1* and various *pTRV2*‐derived vectors (*TRV2*, *V2‐WRKY49‐D2‐1*, *V2‐WRKY49‐D2‐2*, *V2‐AsZAT18‐1*, and *V2‐AsZAT18‐2*) were mixed at a 1:1 ratio in medium containing 19.62 mg L^−1^ acetosyringone (AS), 400 mg L^−1^ cysteine (Cys), and 5 mL L^−1^ Tween‐20. Germinating oat seeds were vacuum‐infiltrated with the mixture at 20 kPa for 5 min, followed by co‐cultivation at 28°C with shaking (180 rpm) overnight. After co‐cultivation, seeds were washed with sterile water to remove surface‐adhered *Agrobacterium* and sown in soil. All primers are listed in Table S20.

### Transcriptional activity assay

The dual‐LUC transcriptional activity assay was performed as previously described (Sun et al., 2019). Tobacco leaves were co‐transfected with *AsWRKY49pro:LUC* reporter constructs and the *AsZAT18–GFP* vector. *35S:REN* was used as an internal control. Total proteins were extracted from the samples using dual‐LUC assay reagents (Coolaber, Beijing, China). The LUC/REN ratio was used to quantify the effect of *AsZAT18* on the promoter activity of *AsWRKY49*, as measured with a GloMax 20/20 luminometer (Promega, Madison, WI, USA). All primers are listed in Table S20.

### Measurement of ion (Na^+^ and K^+^) content

Samples collected for ion content measurement were processed as previously described (Zhang et al., 2025c), and a flame photometer (FP6410, INESA) was used to measure the Na^+^ and K^+^ content.

## CONFLICTS OF INTEREST

The authors declare no conflicts of interest.

## AUTHOR CONTRIBUTIONS

H.D. and Q.S. conceived and designed the project. C.D. analyzed the transcriptome data. Y.Y. and Q.S. performed the experiments. W.L. and X.W. collected the phenotypic data and performed the GWAS analysis. X.L. constructed the transgenic oat plants. M.L., Y.L., S.W., and W.L. assisted with parts of this work. C.D., Y.Y., and Q.S. wrote the manuscript, and H.D. revised it. All authors read and approved of its content.

## Supporting information

Additional Supporting Information may be found online in the supporting information tab for this article: http://onlinelibrary.wiley.com/doi/10.1111/jipb.70171/suppinfo

**Figure S1.** Phenotype and transcriptome analyses of oat under salt stress
**Figure S2.** Details of salt‐responsive transcription factors in oat
**Figure S3.** Differentially expressed gene co‐expression network analysis
**Figure S4.** Salt‐responsive DEGs in the five homologous groups
**Figure S5.** Identification of *AsWRKY49‐D2* by GWAS
**Figure S6.** *AsWRKY49‐D2* positively regulates the salt tolerance in oats
**Figure S7.** *AsZAT18* regulates the expression of *AsWRKY49‐D2*
**Figure S8.** *AsWRKY49‐D2* regulates the expression of *AsSOS2* and *AsSOS3* via binding to their promoters

**Table S1.** Mapping rate of DNA‐Seq and RNA‐seq data to the *A. sativa* genome
**Table S2.** Number of DEGs in each treatment of salt stress
**Table S3.** FPKM of differentially expressed genes
**Table S4.** KEGG function enrichment of DEGs
**Table S5.** GO function enrichment of DEGs
**Table S6.** Gene IDs of transcription factors in the oat genome, with red font indicating transcription factors responsive to salt
**Table S7.** Number of transcription factors and differentially expressed transcription factors in the oat genome
**Table S8.** Gene count in different modules. Red color presents the tissue‐specific module
**Table S9.** KEGG enrichment results of the co‐expression network in different modules
**Table S10.** GO enrichment results of the co‐expression network in different modules
**Table S11.** *SOS1*, *SOS1*, and *SOS3* gene ids in OT3089 that respond to salt. Red color represents the *SOS* genes involved in the Meyellow module
**Table S12.** List of trimeric genes in oats
**Table S13.** List of dominant DETFs in the A subgenome
**Table S14.** List of dominant DETFs in the C subgenome
**Table S15.** List of dominant DETFs in the D subgenome
**Table S16.** List of specific DETFs in the A subgenome
**Table S17.** List of specific DETFs in the C subgenome
**Table S18.** List of specific SRTFs in the D subgenome
**Table S19.** Geographical distribution of different types of oats
**Table S20.** List of primers in the article
