# Supplementary material for: A natural variation within duplicated AsWRKY49‐D2 drives the subgenomic functional divergence of homeologs in salt response of allohexaploid oats
Source: J Integr Plant Biol. 2026 Feb 9;68(5):1367–83. doi: 10.1111/jipb.70171 (PMC13140011; doi:10.1111/jipb.70171)
Supplement: Supplementary file 1 — Figure S1. Phenotype and transcriptome analyses of oat under salt stress Figure S2. Details of salt‐responsive transcription factors in oat Figure S3. Differentially expressed gene co‐expression network analysis Figure S4. Salt‐responsive DEGs in the five homologous groups Figure S5. Identification of AsWRKY49‐D2 by GWAS Figure S6. AsWRKY49‐D2 positively regulates the salt tolerance in oats Figure S7. AsZAT18 regulates the expression of AsWRKY49‐D2 Figure S8. AsWRKY49‐D2 regulates the expression of AsSOS2 and AsSOS3 via binding to their promoters [file JIPB-68-1367-s001.docx]

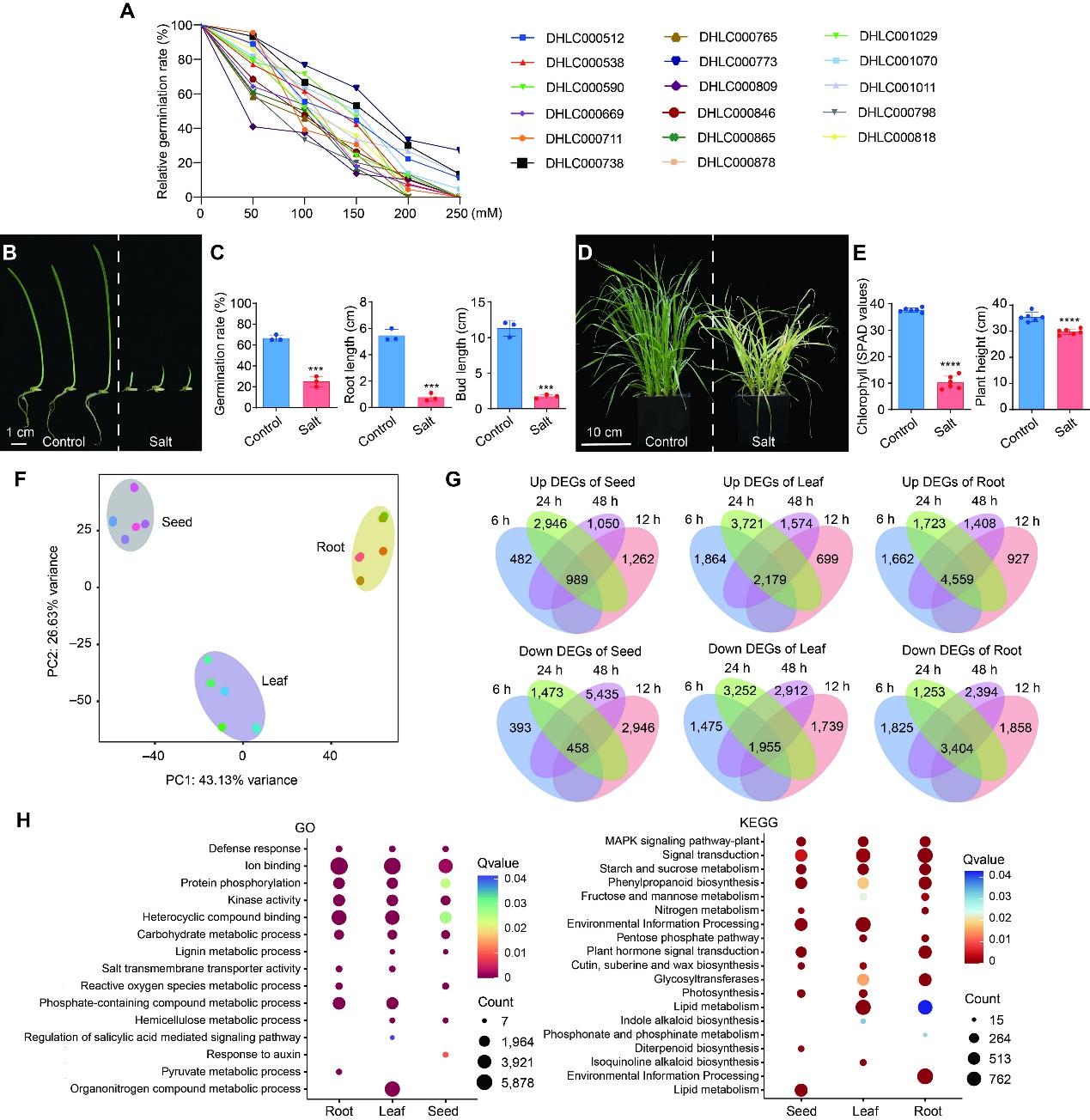


**Figure S1. The phenotype and transcriptome analysis of oat under salt stress.**

**(A)** The relative germination rate of 17 randomly selected oat accessions under different NaCl concentration. 50 seeds were germinated in water and aqueous solutions with different concentrations of NaCl for 7 days, respectively. Relative Germination Rate = (Germination Rate of Treatment Group) / (Germination Rate of Control Group). **(B)** Phenotype of oat seeds at the germination stage under normal and salt stress conditions (200 mM NaCl). **(C)** The germination rate, length of plumules and radicles of oat seeds under normal and salt stress conditions (200 mM NaCl). **(D)** Phenotype of oat seedlings under normal and salt stress conditions. **(E)** The chlorophyll content and plant height of oat seedlings under normal and salt stress conditions. **(F)** PCA of RNA-seq data derived from the 45 oat samples. **(G)** Venn diagram showing the numbers of up-regulated and down-regulated DEGs in each tissue. **(H)** GO and KEGG terms of all DEGs in oat under salt stress.


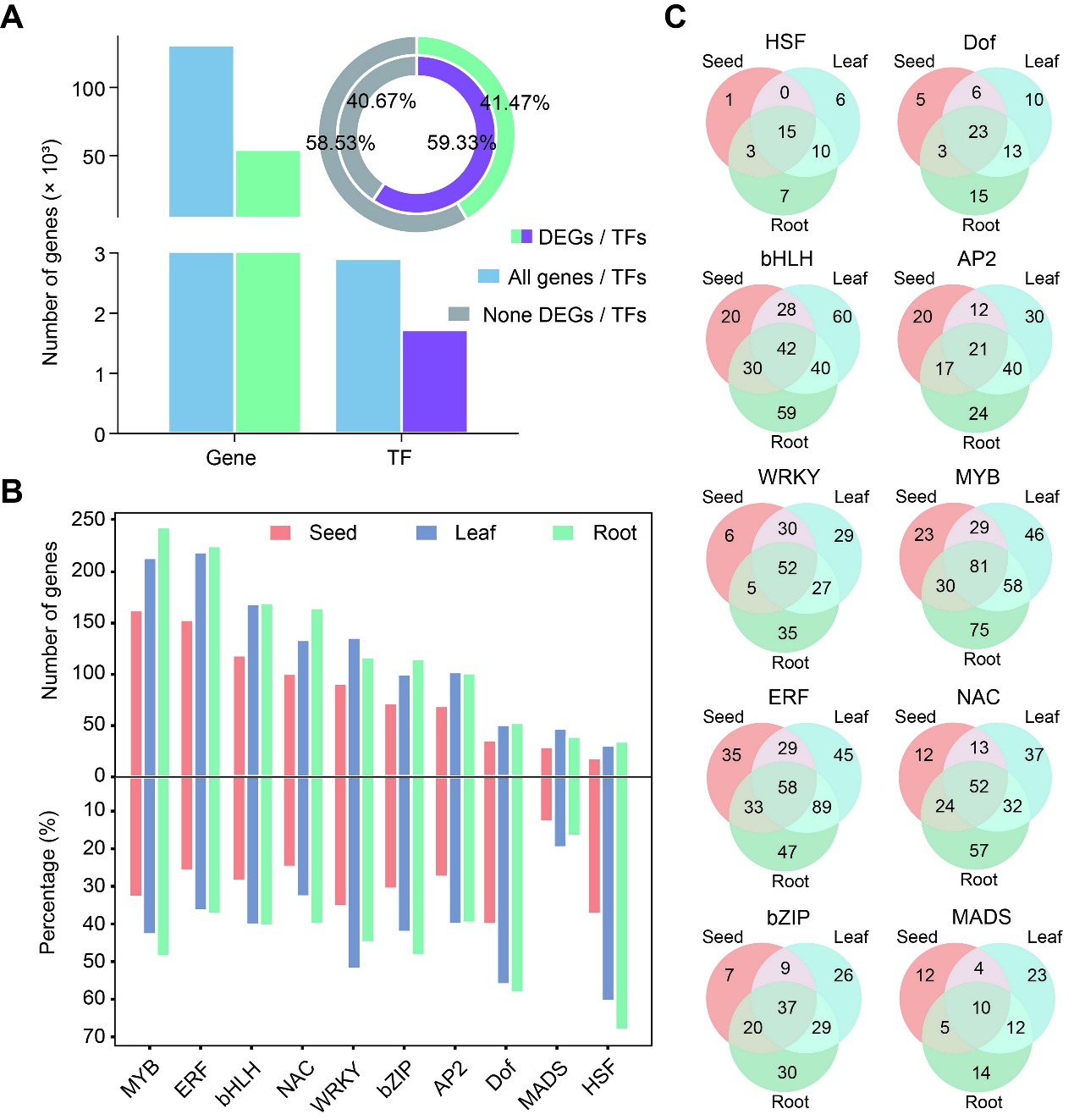


**Figure S2**. **Details of salt-responsive transcription factors in oat.**

**(A)** The number of all differentially-expressed genes and TFs in oat. **(B)** The number and percentage of differentially-expressed TFs in each tissue. **(C)** Venn diagram showing the number of differentially-expressed TFs in each tissue.


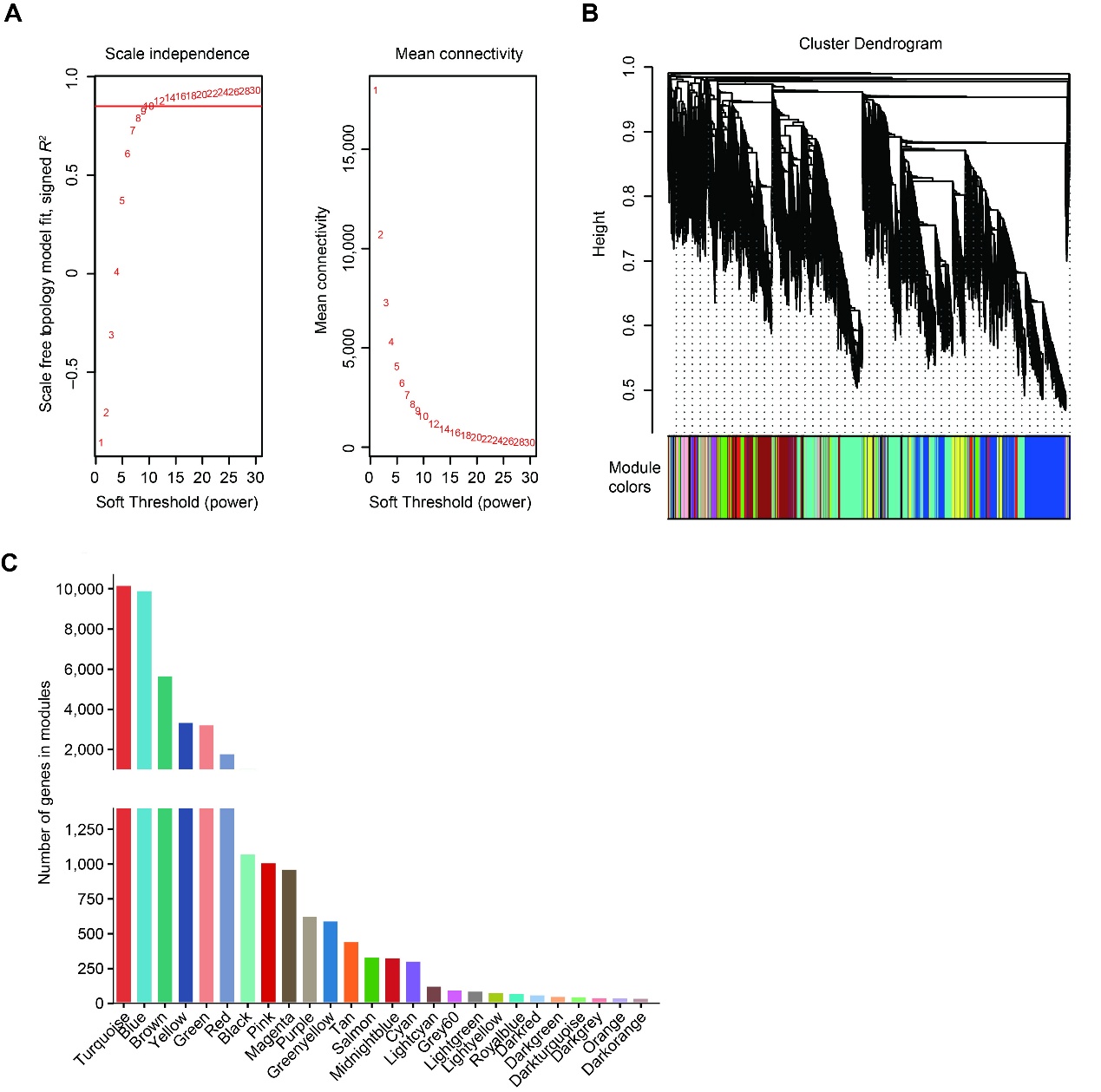


**Figure S3. Differentially expressed genes co-expression network analysis.**

**(A)** Analysis of network topology for various soft thresholding (power). **(B)** Dendrogram showing modules identified by the weighted gene co-expression network analysis (WGCNA) and clustering dendrogram of DEGs. The same color represents the same module. **(C)** The DEG number in each module.


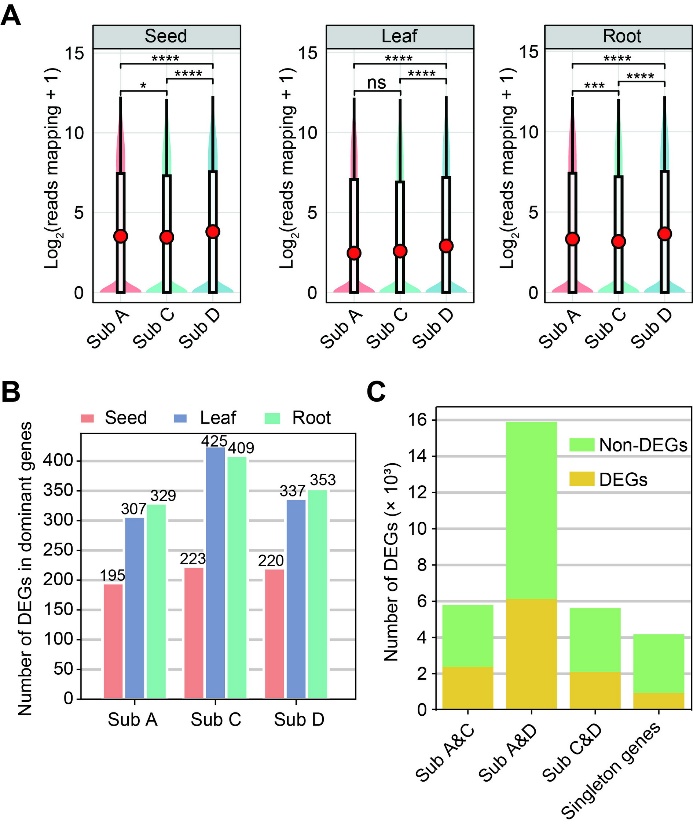


**Figure S4. Salt-responsive DEGs in the five homologous groups.**

**(A)** Expression of all genes in each subgenome of oat across three tissues. Statistical significance was determined by a Student’s *t*-test (**P* < 0.05, ****P* < 0.001, *****P* < 0.0001, ns = not significant). **(B)** The number of dominantly-expressed DEGs in each subgenome of oat across three tissues. **(C)** The number of divergently-expressed DEGs not represented by homologous triads (diads and singletons).


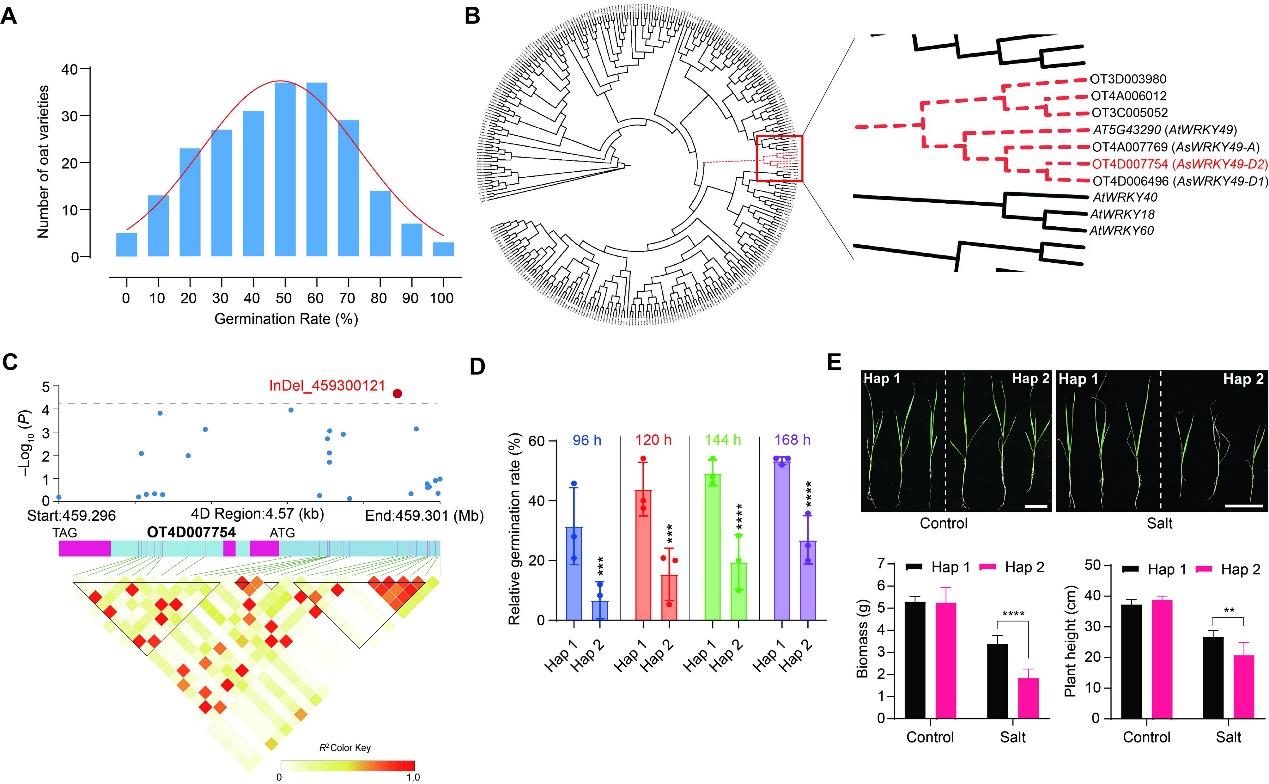


**Figure S5**. **Identification of *AsWRKY49-D2* by GWAS**

**(A)** Frequency distribution histogram of germination rate among 225 oat accessions, with the red curve representing kernel density estimation. **(B)** Phylogenetic analysis of *AsWRKY49-D2* with its homologs from *Arabidopsis*. **(C)** The association analysis of the genetic variations in *OT4D007754* with germination rate among 225 oat accessions. Upper panel shows the characteristics of variations significantly associated with germination rate, with the red dot highlighting the most significantly associated structural variation (InDel_459300121); lower panel displays the pairwise linkage disequilibrium (LD) pattern of these variations. **(D)** Relative germination rates of randomly selected Hap1 and Hap2 oat accessions at 96 h, 120 h, 144 h, and 168 h after salt treatment. Data are presented as mean ± *SD* from three independent experiments; ****P* < 0.001, *****P* < 0.0001 (Student’s t-test). **(E)** Top panel: Phenotypes of randomly selected Hap1 and Hap2 oat accessions under control and salt stress. Photographs were taken under normal conditions and 10 days after salt treatment. Bottom panel: Biomass and plant height of the plants displayed in **(E)**. Data are mean ± *SD*; ***P* < 0.01, *****P* < 0.0001 (Student’s t-test).


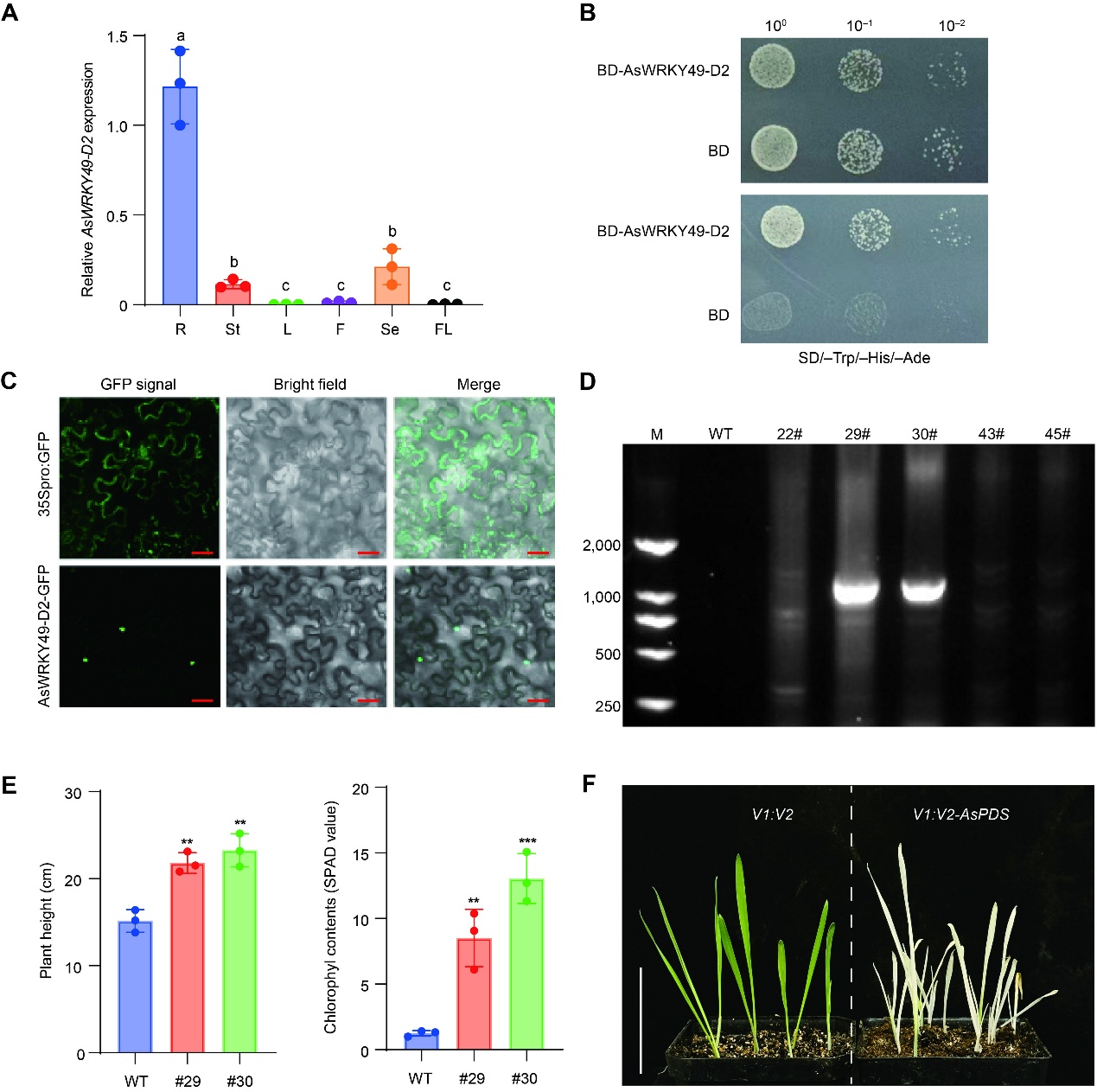


**Figure S6. *AsWRKY49-D2* positively regulates the salt tolerance in oats**

**(A)** Relative expression level of *AsWRKY49-D2* in various oat tissues. R, root; St, stem; L, leaf; F, flower; Se, seed; FL, flag leaf. Data represent mean ± *SD* with *n* = 3, and different letters indicate the significant differences at the 0.05 level determined by one-way ANOVA test. **(B)** Trans-activation activity analysis of AsWRKY49-D2. **(C)** Subcellular localization of AsWRKY49-D2 in *N. benthamiana* leaves. Scale bar = 50 μm. **(D)** PCR verification of *AsWRKY49-D2* overexpression lines. The primers used in the PCR system are derived from the vector and the target sequence, respectively. **(E)** Plant height and chlorophyll content in wild-type (WT) plants and *AsWRKY49-D2* overexpression plants. Values are means ± *SD* from at least three independent experiments; statistical significance was determined by a Student’s *t*-test (***P* < 0.01, ****P* < 0.001). **(F)** Phenotype of *AsPDS* gene knockdown lines established by the TRV-VIGS system. Scale bar = 5 cm.


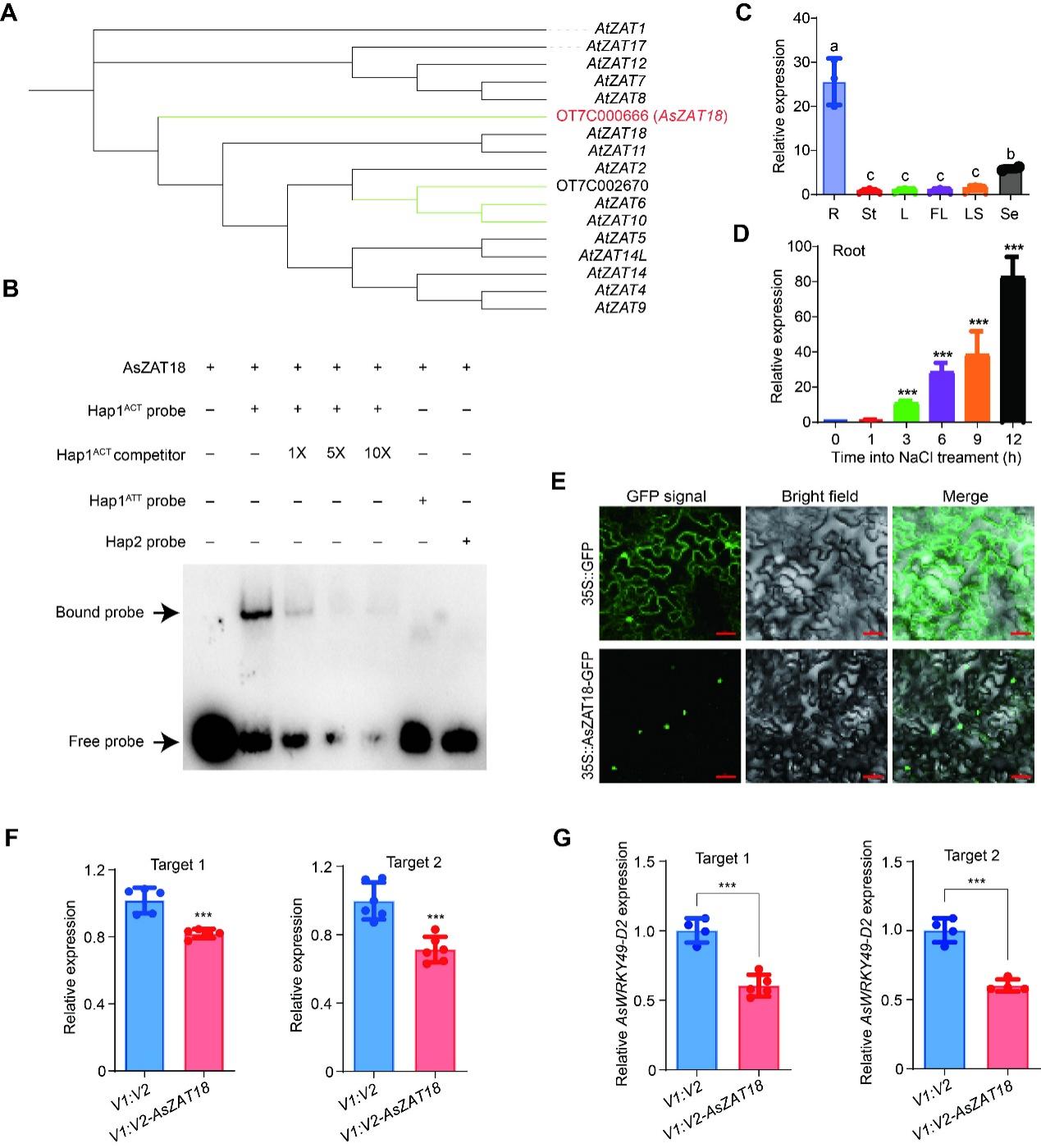


**Figure S7. AsZAT18 regulates the expression of *AsWRKY49-D2***

**(A)** Phylogenetic analysis of *AsZAT18* with its homologs from *Arabidopsis*. **(B)** EMSA assay showing that AsZAT18 directly binds to the Hap1^ACT^ probe. **(C)** Relative expression of *AsZAT18* in various oat tissues. R, root; St, stem; L, leaf; FL, flag leaf; LS, leaf sheath; Se, seed. Data represent mean ± *SD* with *n* = 3, and different letters indicate the significant differences at the 0.05 level determined by one-way ANOVA test. **(D)** Relative expression of *AsZAT18* in roots at different time following salt treatment. **(E)** Subcellular localization of AsZAT18. Scale bar = 50 μm. **(F)** The expression of *AsZAT18* in gene knockdown lines generated by TRV-VIGS system. **(G)** The expression of *AsWRKY49-D2* in the root of *AsZAT18*-knockdown plants. Statistical significance in **(C)**, **(E)**, and **(F)** was determined by a Student’s *t*-test (****P* < 0.001).


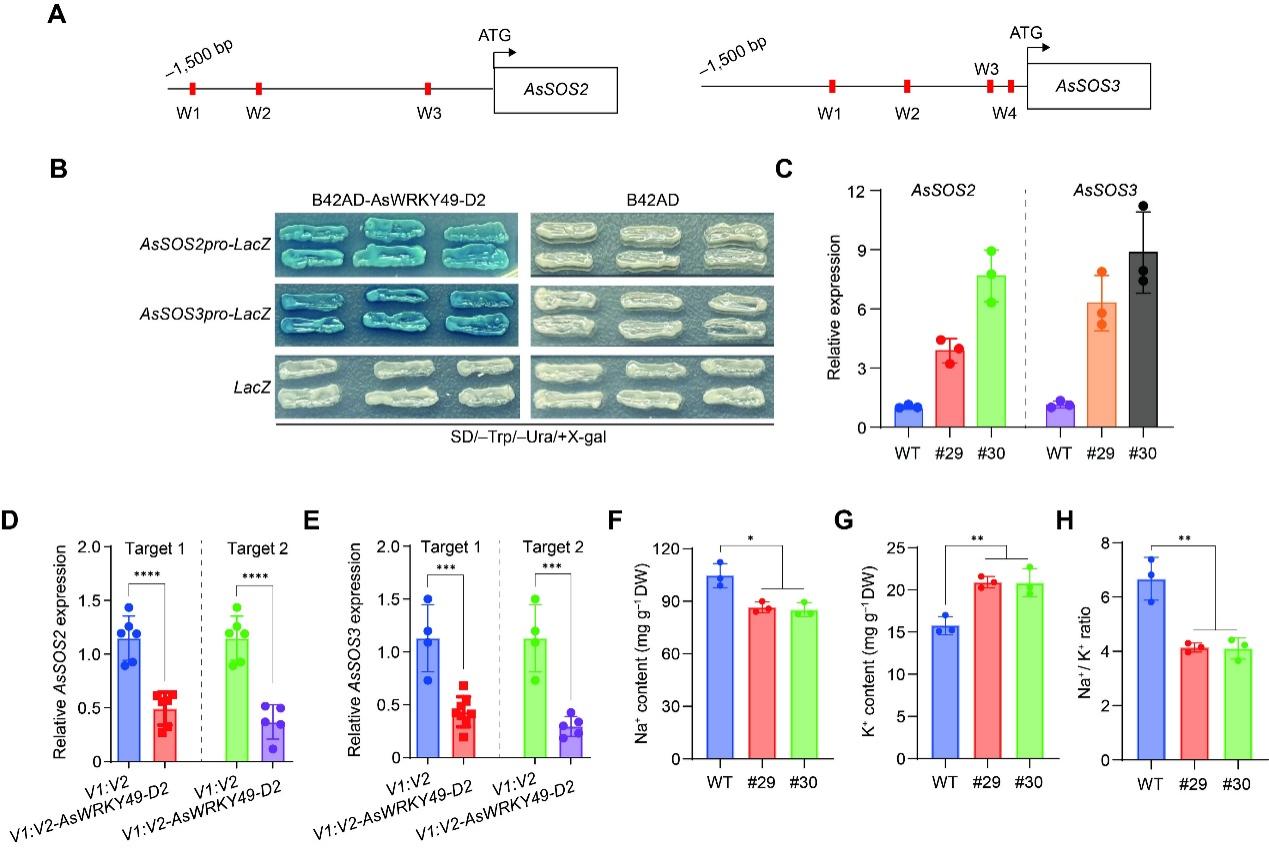


**Figure S8.** **AsWRKY49-D2 regulates the expression of *AsSOS2* and *AsSOS3* via binding to their promoters**

**(A)** Schematic diagrams of *AsSOS2* and *AsSOS3* promoter regions. The red box presents the conserved W-box motif. **(B)** Results of yeast one-hybrid (Y1H) assay showing the binding AsWRKY49-D2 to the promoters of *AsSOS2* and *AsSOS3*. *B42AD* and *LacZ* empty vectors are served as negative controls. **(C)** The expression of *AsSOS2* and *AsSOS3* in *AsWRKY49-D2* overexpression plants. **(D**–**E)** The expression of *AsSOS2* and *AsSOS3* in *V1:V2-AsWRKY49-D2* gene knockdown plants. **(F**–**H)** The Na^+^ content **(F)**, K^+^ content **(G)**, and Na^+^/K^+^ ratio **(H)** in the shoot tissues of wild type and *AsWRKY49-D2* overexpression plants following treatment with 200 mM NaCl. Statistical significance in **(D**–**H)** was determined by a Student’s *t*-test (**P* < 0.05, ***P* < 0.01, ****P* < 0.001, *****P* < 0.0001).
